# Machine learning-driven development of a disease risk score for COVID-19 hospitalization and mortality: a Swedish and Norwegian register-based study

**DOI:** 10.3389/fpubh.2023.1258840

**Published:** 2023-12-07

**Authors:** Saeed Shakibfar, Jing Zhao, Huiqi Li, Hedvig Nordeng, Angela Lupattelli, Milena Pavlovic, Geir Kjetil Sandve, Fredrik Nyberg, Björn Wettermark, Mohammadhossein Hajiebrahimi, Morten Andersen, Maurizio Sessa

**Affiliations:** ^1^Department of Drug Design and Pharmacology, Pharmacovigilance Research Center, University of Copenhagen, Copenhagen, Denmark; ^2^Department of Drug Design and Pharmacology, Drug Safety Group, University of Copenhagen, Copenhagen, Denmark; ^3^Department of Pharmacy, Pharmacoepidemiology and Drug Safety Research Group, Faculty of Mathematics and Natural Sciences, University of Oslo, Oslo, Norway; ^4^UiO: RealArt Convergence Environment, University of Oslo, Oslo, Norway; ^5^School of Public Health and Community Medicine, Institute of Medicine, Sahlgrenska Academy, University of Gothenburg, Gothenburg, Sweden; ^6^Department of Informatics, Faculty of Mathematics and Natural Sciences, University of Oslo, Oslo, Norway; ^7^Department of Pharmacy, Pharmacoepidemiology and Social Pharmacy, Uppsala University, Uppsala, Sweden

**Keywords:** COVID-19, machine learning, disease risk score, prediction modeling, artificial intelligence

## Abstract

**Aims:**

To develop a disease risk score for COVID-19-related hospitalization and mortality in Sweden and externally validate it in Norway.

**Method:**

We employed linked data from the national health registries of Sweden and Norway to conduct our study. We focused on individuals in Sweden with confirmed SARS-CoV-2 infection through RT-PCR testing up to August 2022 as our study cohort. Within this group, we identified hospitalized cases as those who were admitted to the hospital within 14 days of testing positive for SARS-CoV-2 and matched them with five controls from the same cohort who were not hospitalized due to SARS-CoV-2. Additionally, we identified individuals who died within 30 days after being hospitalized for COVID-19. To develop our disease risk scores, we considered various factors, including demographics, infectious, somatic, and mental health conditions, recorded diagnoses, and pharmacological treatments. We also conducted age-specific analyses and assessed model performance through 5-fold cross-validation. Finally, we performed external validation using data from the Norwegian population with COVID-19 up to December 2021.

**Results:**

During the study period, a total of 124,560 individuals in Sweden were hospitalized, and 15,877 individuals died within 30 days following COVID-19 hospitalization. Disease risk scores for both hospitalization and mortality demonstrated predictive capabilities with ROC-AUC values of 0.70 and 0.72, respectively, across the entire study period. Notably, these scores exhibited a positive correlation with the likelihood of hospitalization or death. In the external validation using data from the Norwegian COVID-19 population (consisting of 53,744 individuals), the disease risk score predicted hospitalization with an AUC of 0.47 and death with an AUC of 0.74.

**Conclusion:**

The disease risk score showed moderately good performance to predict COVID-19-related mortality but performed poorly in predicting hospitalization when externally validated.

## 1 Introduction

Severe acute respiratory syndrome coronavirus 2 (SARS-CoV-2) has had a profound impact on global health, the economy, and education (1). Mitigation/containment strategies and vaccination programs have been designed to reduce the incidence of coronavirus disease 2019 (COVID-19), to prevent major surges of patients being hospitalized, to protect vulnerable populations with a high risk of severe illness or poor prognosis, and to save lives (2–5). Neither vaccines nor mitigation or containment strategies have been fully able to prevent the transmission of SARS-CoV-2 and the development of severe illness or death from COVID-19. Important reasons are the unavailability of vaccines that confer 100% protection (6, 7), emerging SARS-CoV-2 variants (8, 9), and the uncertainty of which individuals are at higher risk of severe COVID-19 or poor prognosis (10–15).

Understanding the heterogeneity in risk of severe COVID-19 and identifying patients with poor prognosis has been a global public health priority since the pandemic started, as it was quickly understood that identifying risk factors is crucial to contextualize the response and focus resources and mitigation and containment strategies (16). Prognostic tools for the prediction of COVID-19 disease severity or poor prognosis have been extensively developed. However, so far, achievements have been limited, as available prediction tools showed a lack of robustness and generalizability in performance across populations and settings (17).

Heterogeneity of populations and risk factors across geographical settings (10), including the effects of social determinants and their interplay (14) and the lack of validation of prognostic tools in multiple cohorts (17) has played a key role for lack of robustness and generalizability in performance across populations and settings. We recently conducted a systematic review and found that previous machine learning and artificial intelligence (AI)-based predictive models for COVID-19 hospitalization and mortality were affected by a high risk of bias or lack of applicability, especially due to lack of external validation of prognostic models (18). Of note, there are examples of studies that have developed AI-driven models for COVID-19 hospitalization or death, which underwent external validation (19, 20). However, it is worth mentioning that we consider these studies as having a high risk of bias (18). Therefore, this study aimed at overcoming the limitations of the previously developed AI models by more stringently identifying predictors of COVID-19 severity and using them to develop a disease risk score (DRS) for COVID-19-related hospitalization and for COVID-19 death – overall and across the COVID-19 waves – for residents in Sweden, and externally validate the DRS in Norway.

## 2 Methods

### 2.1 Study design and setting

This is a population-based study including all residents in Sweden from November 2019 and from February 2020 in Norway who tested positive for SARS-CoV-2 infection by real-time polymerase chain reaction (RT-PCR) up to the latest available data (August 2022 in Sweden and December 2021 in Norway).

### 2.2 Study population

The source populations were 2.6 million in Sweden and 0.4 million in Norway. From the source populations, we identified individuals that were admitted to hospital for COVID-19 as primary diagnosis (International Classification of Diseases version 10, ICD-10: U07) up to 14 days after the positive test (i.e., cases) as done by prior research (21). Among the cases, we further identified fatal cases who died within 30 days of COVID hospitalization. For cases, the date of hospitalization was defined as the index date. Up to five individuals per case were randomly selected as controls among those eligible in the study population and in the risk set on the case index date, matched by year of birth and sex, and who at the time of the matching had not emigrated, and were not hospitalized, and had not died of SARS-CoV-2 infection.

### 2.3 Data sources

#### 2.3.1 Sweden

The Swedish data originated from the SCIFI-PEARL (Swedish COVID-19 Investigation for Future Insights – a Population Epidemiology Approach using Register Linkage) project (22), which has expanded to include all individuals in the Swedish population (approximately 10.2 million inhabitants) and is being updated regularly. The national database of notifiable diseases (Sminet) was used to identify positive SARS-CoV-2 RT-PCR test results (22). The Swedish National Patient Register and the Cause-of-Death Register were used to identify individuals that were hospitalized and subsequently died (22, 23). Data from the National Prescribed Drug Register and the Swedish National Patient Register were used to identify predictors. The unique identification number assigned to Swedish residents was used to link individual records across these registers (24), and the database was then pseudonymized. The data from the National Patient Register and Cause-of-Death Register was available from 1 January 2015 and onward, while data from the National Prescribed Drug Register from 1 January 2018 and onward. In Swedish registers, due to the restrictions of health data, ICD-10 codes in the Swedish data are of varied levels of detail (Supplementary Table 1). Anatomical Therapeutic Chemical Classification (ATC) codes in the Swedish data are also of varied levels (Supplementary Table 2).

#### 2.3.2 Norway

The Norwegian data sources included healthcare registries covering the entire Norwegian population, approximately 5.5 million inhabitants. Specifically, the Norwegian Surveillance System for Communicable Diseases (MSIS) (25) was used to obtain information on notified infectious diseases including SARS-Cov-2, and the Norwegian Patient Registry (NPR) (19, 20) to identify individuals hospitalized for COVID-19. Mortality was assessed in the Norwegian Cause of Death Registry (26). The Norwegian Prescription Database (NorPD) (27) and the NPR were used to identify predictors. Similar to Sweden, due to the data minimization policy, the ICD-10 codes from the NPR are of level-3, except for diagnostic codes for COVID-19 which are of full length. The COVID-19 ICD-10 codes used for this study is U07.1 and U07.2. The ATC codes from Norwegian data are of level-5.

### 2.4 Candidate empirical covariates for COVID-19-related hospitalization and mortality

Two different covariate assessment windows were used to generate the high-dimensional set of variables further used in machine learning models to develop the DRS for COVID-19 hospitalization and mortality. We identified dispensed prescriptions for medicine in Sweden and Norway using a covariate assessment window of 365 days before the index date, while for diagnoses and surgery/procedures from hospital inpatient admissions and specialist outpatient visits, we used all the information available in Sweden and Norway before the index date.

We did not set rules for the granularity regarding ICD-10 codes or the ATC codes, as the data from Sweden and Norway did not have homogeneous granularity to set such rules.

Within each of the *p* data dimensions (i.e., inpatient/outpatient diagnostic codes, procedures/surgeries, and drugs dispensed) codes were sorted by their prevalence. Prevalence was measured as the code period prevalence, i.e., the proportion of individuals having a specific code at least once during the covariate assessment windows. The most prevalent codes were identified as candidate empirical covariates in each data dimension and we assessed how frequently those codes were recorded for each patient during the covariate assessment windows. We created three binary variables for each code: code occurred 1 time (no/yes), code occurred more than the median number of times, and code occurred more than the 75th percentile number of times. A code that appeared above the 75th percentile number of times would have a true value for all three occurrence variables. Therefore, three covariates (code occurring 1 time, median number of times, and 75th percentile number of times) were generated for each ICD-10/ATC code.

### 2.5 Data analysis

#### 2.5.1 Filtering and prioritization of candidate empirical covariates

The first filtering approach was based on variance. The total list of features generated using the approach described in section 2.4 was screened and variables having ≥95% identical values across individuals in the study population were removed. Then, an ensemble feature selection (EFS) approach (28) was implemented to rank features’ importance for COVID-19 hospitalization and death with the final goal of prioritizing the most important predictors for these outcomes. EFS incorporates six different feature prioritization methods for binary classifications, namely:

(1) *p*-value from the Mann–Whitney-U Test of being classified as being or not being hospitalized or dying for COVID-19.
(2) and (3) *p*-value from the Pearson and Spearman correlation analysis based on relevance and redundancy according to Yu and Liu (29).
(4) β-coefficients from a logistic regression of Z-transformed predictors.
(5) Area under receiver operating characteristic curve (AUC)-based variable importance measure from ensembles of multiple decision trees based on the random forest algorithm according to Breiman et al. (30).
(6) AUC-based variable importance measure from ensembles of multiple decision trees based on the Gini impurity index (31).

The results of each feature prioritization method were normalized. The normalized ensemble score of the 6 prioritization methods was used for ranking features’ importance which was then used to identify the optimal number of features. The optimal number of features was identified by looking at the deviation of the AUC, which was perfectly correlated with the deviation of the ensemble score, which was computed by sequentially including the top-ranked features for predicting COVID-19 hospitalization or mortality one at a time. Specifically, if the standard deviation of the further improvements of the AUC was less than 0.0035, we stopped adding more predictors. In order to build a parsimonious model, we computed the standard deviation of the AUC each time a new feature was included. We stopped including predictors if the standard deviation of the further improvement from the next model turned out to be less than 0.0035. We chose 0.0035 because it empirically appeared to be the largest hence optimal value. In other words, when the standard deviation of the subsequent AUC improvements is less than 0.0035, the improvement of the performance of expanded models was either negligible or negative. Then, we used 3 commonly used machine learning classification models to incorporate the prioritized set of features: random partitioning, ranger random forest, and logistic regression from the R package *caret* versions 6.0–93 (Sweden) and 6.0–91 (Norway) (32). The prioritized features were used to develop the DRS (section 2.5.2). The machine learning model with the best performance was used for the prediction of COVID-19 hospitalization and mortality using the DRS. All analyses were conducted using the R versions 4.2.2 (Sweden) and 4.1.3 (Norway) (33).

#### 2.5.2 Disease risk score

After identifying the optimal number of predictors, we applied the following formula to obtain weights for each selected predictor that were > 0 (Formula 1). This formula was previously validated in a similar research context (34).

$\begin{array}{l} \mathrm{weights}=\mathrm{normalized}\;\mathrm{ensemble}\;\mathrm{score}\;+\;2^{*}|\min\\ \;\left(\mathrm{normalized}\;\mathrm{ensamble}\;\mathrm{score}\right)\; \end{array}$

Formula 1. Development of the weights using the normalized ensemble score.

By applying the weights to each predictor, we calculated the DRS for each individual and we used it to calculate the predicted probability of developing the outcomes. The probability of developing the outcomes was based on the DRS by average AUC based on a 5-fold cross-validation (explained in section 2.5.3) using only one control per case to avoid an unbalanced performance matrix. This choice was crucial as we did not’ rely solely on AUC as a performance metric; thus, utilizing a balanced dataset was important.

Calibration was performed to get bias-corrected (overfitting-corrected) estimates of predicted probabilities using the DRS.

#### 2.5.3 Models performance

To estimate the models’ performance and to avoid any overfitting problem of benchmarked classification models, a 5-fold cross-validation method was applied. Finally, overall model performance was assessed by averaging model performances for each fold of the cross-validation. For assessing the model performance, the accuracy, AUC, sensitivity, and specificity were measured for all models using a confusion matrix (35). The gold standard in this analysis was the hospitalization/death record in Swedish or Norwegian registers and we compared the gold standard with the prediction from the models.

#### 2.5.4 External validation

The DRS developed using the Swedish data was externally validated in Norway and the model’s performance in Norwegian data was assessed according to approaches described in section 2.5.3.

#### 2.5.5 Stratified analysis by COVID-19’s waves & patients’ age and sex

The approaches described in sections 2.5.1–2.5.3 were also performed separately within data from the COVID-19 waves. Although there is no formal epidemiological definition of a wave of infection, for SARS-CoV-2 it has been characterized as ‘a rising number of sick individuals, a defined peak and then a decline’; this was the working definition of a wave of infection for our study (36). According to this definition, 3 waves were identified in Sweden and Norway during the study period (Supplementary Figure 1). Variability between Sweden and Norway regarding the time of onset of waves of COVID-19 disease (and therefore hospitalizations and mortality) has been observed and described in the scientific literature (36). Therefore, we have used a different period for each wave in Sweden and Norway (Supplementary Table 3).

Additionally, the approaches described in sections 2.5.1–2.5.3 were performed separately by age group ([0–18), (18–65)), [65–75), [75-maximum age in the data] and sex (Male, Female).

#### 2.5.6 Descriptive analysis

We performed a descriptive analysis by providing summary tables with information on the age and sex of cases and controls for the overall period and separately by waves. Additionally, we tabulated and plotted the featured predictors (including their predicted probability, weights, and prevalence), the models’ performance, and the deviation of the ensemble score to identify the optimal set of predictors for each study outcome – for the overall period and separately by waves, age and sex. Pairwise correlation plots visualize the correlation between the DRS, prevalence, weights, and the probability of the outcome separately for hospitalization and mortality, overall and stratified by waves, age group, and sex. Fisher and chi-square tests were used to calculate *p*-values for descriptive statistics.

#### 2.5.7 Reporting guidelines and bias assessment

To develop our prediction model, we followed a rigorous methodology in accordance with the TRIPOD guidelines (37). To assess bias in our prediction model, we followed the domain-specific criteria outlined in the PROBAST guidelines (38). Firstly, we evaluated the participant selection process for potential biases, considering factors such as sampling methods, inclusion/exclusion criteria, and representativeness of the study population. Secondly, we examined the predictor variables to ensure they were measured accurately, avoiding any potential bias due to measurement errors or missing. Similarly, we assessed the outcome measurement process, considering any potential biases that could arise from misclassification or measurement variability.

## 3 Results

### 3.1 Demographic characteristics

**Figure 1:**
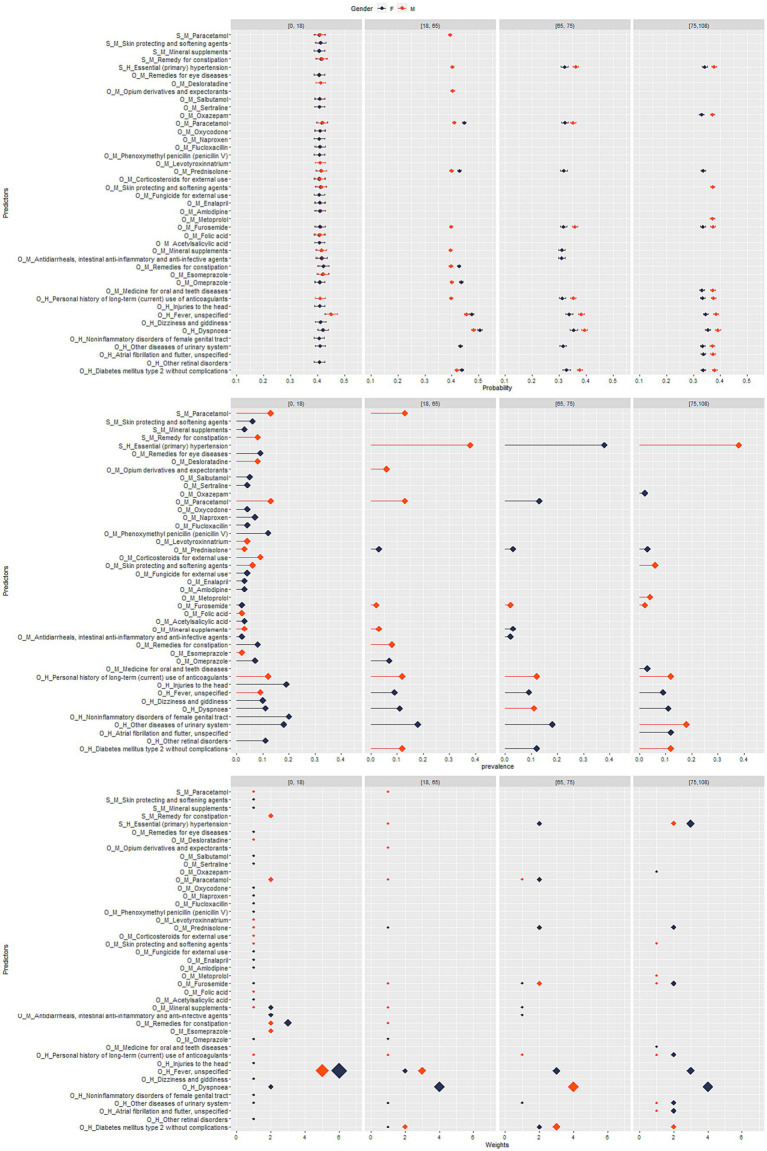
*Sweden:* stratified analysis by sex and age group for top-ranked predictors, their prevalence, weight, and predicted probability for COVID-19 hospitalization.

**Figure 2:**
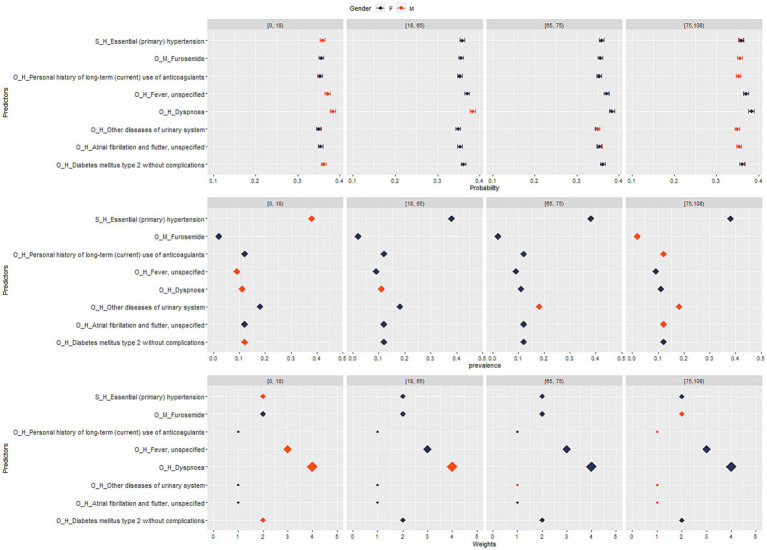
*Sweden:* stratified analysis by sex and age group for top-ranked predictors, their prevalence, weight, and predicted probability for COVID-19 death.

In our study population of cases and selected controls, across Sweden and Norway, there were 124,560 out of 538,277 (23.1%) and 10,835 out of 53,744 (20.2%) individuals hospitalized for COVID-19, respectively. In total, 15,877 (2.9%) and 928 (1.7%) died within 30 days following COVID-19 hospitalization in Sweden and Norway, respectively. Demographic characteristics of hospitalized cases and selected controls in Sweden, including age and sex, are provided in Table 1 and Supplementary Table 4 overall and separately by waves, for Norway and Sweden, respectively. There was a significantly higher mortality and hospitalization for COVID-19 among men (value of *p* <0.001) (Figures 1, 2). Younger patients showed a fewer hospitalization for COVID-19 (for all comparisons among age groups, value of *p* < 0.001) (Figure 1). These findings remained consistent in the external validation set (Supplementary Figure 2) and across multiple waves of data collection, indicating the robustness and reliability of the observed trends (for all comparisons among age groups and sex across waves, value of *p* < 0.05).

**Table 1:** Age and sex distribution of cases (COVID-19 hospitalization and COVID-19 death) and respective selected age/sex-matched controls among COVID-19 test-positive individuals in Sweden January 2020 to August 2021.

Hospitalization						Mortality			
Waves	Variable	Group	Control	Cases	Overall	Group	Control	Cases	Overall
Overall (%)			**N = 413,717**	**N = 124,560**	**N = 538,277**		**N = 88,388**	**N = 15,877**	**N = 104,265**
	Age – group 1	[0, 18)	15,593 (3.8)	5,102 (4.1)	20,695 (3.8)	[0, 18)	140 (0.2)	24 (0.2)	164 (0.2)
	Age – group 2	[18, 65)	276,472 (66.8)	63,579 (51.0)	340,051 (63.2)	[18, 65)	19,114 (21.6)	1,372 (8.6)	20,486 (19.6)
	Age – group 3	[65, 75)	52,744 (12.7)	18,837 (15.1)	71,581 (13.3)	[65, 75)	21,052 (23.8)	2,567 (16.2)	23,619 (22.7)
	Age – group 4	[75, 108]	68,908 (16.7)	37,042 (29.7)	105,950 (19.7)	[75, 108]	48,082 (54.4)	11,914 (75.0)	59,996 (57.5)
	Sex	Male	202,115 (48.9)	65,667 (52.7)	267,782 (49.7)	Male	51,528 (58.3)	9,331 (58.8)	60,859 (58.4)
		Female	211,602 (51.1)	58,893 (47.3)	270,495 (50.3)	Female	36,860 (41.7)	6,546 (41.2)	43,406 (41.6)
**Wave 1 (%)**			**N = 51,878**	**N = 20,856**	**N = 72,734**		**N = 20,169**	**N = 3,994**	**N = 24,163**
	Age – group 1	[0, 18)	921 (1.8)	265 (1.3)	1,186 (1.6)	[0, 18)	13 (0.1)	3 (0.1)	16 (0.1)
	Age – group 2	[18, 65)	36,195 (69.8)	9,556 (45.8)	45,751 (62.9)	[18, 65)	5,254 (26.0)	415 (10.4)	5,669 (23.5)
	Age – group 3	[65, 75)	6,352 (12.2)	3,461 (16.6)	9,813 (13.5)	[65, 75)	4,731 (23.5)	684 (17.1)	5,415 (22.4)
	Age – group 4	[75, 108]	8,410 (16.2)	7,574 (36.3)	15,984 (22.0)	[75, 108]	10,171 (50.4)	2,892 (72.4)	13,063 (54.1)
	Sex	Male	26,283 (50.7)	11,698 (56.1)	37,981 (52.2)	Male	11,811 (58.6)	2,340 (58.6)	14,151 (58.6)
		Female	25,595 (49.3)	9,158 (43.9)	34,753 (47.8)	Female	8,358 (41.4)	1,654 (41.4)	10,012 (41.4)
**Wave 2 (%)**			**N = 188,245**	**N = 59,356**	**N = 247,601**		**N = 56,988**	**N = 8,334**	**N = 65,322**
	Age – group 1	[0, 18)	3,449 (1.8)	1,195 (2.0)	4,644 (1.9)	[0, 18)	61 (0.1)	13 (0.2)	74 (0.1)
	Age – group 2	[18, 65)	131,232 (69.7)	30,783 (51.9)	162,015 (65.4)	[18, 65)	11,051 (19.4)	690 (8.3)	11,741 (18.0)
	Age – group 3	[65, 75)	25,988 (13.8)	9,886 (16.7)	35,874 (14.5)	[65, 75)	13,873 (24.3)	1,365 (16.4)	15,238 (23.3)
	Age – group 4	[75, 108]	27,576 (14.6)	17,492 (29.5)	45,068 (18.2)	[75, 108]	32,003 (56.2)	6,266 (75.2)	38,269 (58.6)
	Sex	Male	97,249 (51.7)	32,557 (54.9)	129,806 (52.4)	Male	33,516 (58.8)	4,887 (58.6)	38,403 (58.8)
		Female	90,996 (48.3)	26,799 (45.1)	117,795 (47.6)	Female	23,472 (41.2)	3,447 (41.4)	26,919 (41.2)
**Wave 3 (%)**			**N = 34,055**	**N = 8,359**	**N = 42,414**		**N = 5,767**	**N = 724**	**N = 6,491**
	Age – group 1	[0, 18)	1,754 (5.2)	553 (6.6)	2,307 (5.4)	[0, 18)	14 (0.2)	1 (0.1)	15 (0.2)
	Age – group 2	[18, 65)	26,641 (78.2)	5,714 (68.4)	32,355 (76.3)	[18, 65)	855 (14.8)	87 (12.0)	942 (14.5)
	Age – group 3	[65, 75)	2,972 (8.7)	864 (10.3)	3,836 (9.0)	[65, 75)	1,365 (23.7)	146 (20.2)	1,511 (23.3)
	Age – group 4	[75, 108]	2,688 (7.9)	1,228 (14.7)	3,916 (9.2)	[75, 108]	3,533 (61.3)	490 (67.7)	4,023 (62.0)
	Sex	Male	16,425 (48.2)	4,149 (49.6)	20,574 (48.5)	Male	3,640 (63.1)	432 (59.7)	4,072 (62.7)
		Female	17,630 (51.8)	4,210 (50.4)	21,840 (51.5)	Female	2,127 (36.9)	292 (40.3)	2,419 (37.3)

Wave 1: 15 February 2020–26 June 2020; Wave 2: 05 November 2020–24 June 2021; Wave 3: 25 June 2021–31 December 2021.

### 3.2 Filtering and prioritization of candidate empirical covariates

In total, there were 23,607 candidate empirical covariates generated during the data management phase for Swedish data sources, and the number of variables after filtering by variance was reduced to 69 (Supplementary Table 5). Plots of the ensemble score along with the deviation of the ensemble score when top-ranked predictors were sequentially included are provided in Supplementary Figures 3–10.

The most important predictors for COVID-19 hospitalization and mortality during the study period and for the different waves are provided in Tables 2, 3. Signs and symptoms like dyspnea and fever emerged as key predictors of hospitalization and death, both overall and across all waves. Chronic conditions such as type 2 diabetes and atrial fibrillation were identified as predictors for COVID 19-related hospitalization and death. Several drugs were also identified as predictors, which are likely to be proxies for diseases (Tables 2, 3). For example, individuals with diabetes (for which metformin is commonly prescribed) or cardiovascular disorders (for which drugs like enalapril, amlodipine, bisoprolol, and metoprolol are used) were found to have a higher risk of severe COVID-19 outcomes. Similarly, individuals with respiratory conditions (for which drugs for inhalation like prednisolone, bronchodilators, and expectorants were used) were found to be associated with a higher risk of hospitalization and/or death.

**Table 2:** Candidate empirical covariates used for the development of the disease risk score for COVID-19 hospitalization in Sweden.

Overall		Wave 1		Wave 2		Wave 3	
Weights	Predictors	Weights	Predictors	Weights	Predictors	Weights	Predictors
5		5	*Hospital admission:* Dyspnea, Fever	5		5	*Hospital admission:* Dyspnea
3	*Hospital admission:* Dyspnea, Fever	3	*Drugs*: Paracetamol	3	*Hospital admission:* Dyspnea	3	*Hospital admission:* Fever *Drugs*: Paracetamol
2		2	*Hospital admission:* Type 2 diabetes, Unspecified tubal pregnancy, Unspecified atrial fibrillation, atrial flutter *Drugs*: Furosemide, Acetylsalicylic acid	2		2	*Hospital admission:* Type 2 diabetes, Unspecified tubal pregnancy *Drugs*: Prednisolone, Furosemide, Omeprazole, Drugs for constipation, Acetylsalicylic acid
1	*Hospital admission:* Type 2 diabetes, Unspecified tubal pregnancy, Personal history of long-term (current) use of anticoagulants *Drugs*: Paracetamol (reimbursed)	1	*Hospital admission:* Personal history of long-term (current) use of anticoagulants, Age-related incipient cataract, Urinary infection, Presence of intraocular lens, Other retinal disorders, Cataract *Drugs*: Metformin, Emollients and protectives, Enalapril, Bisoprolol, Omeprazole, Zopiclone, Apixaban, Oxycodone, Metoprolol, Paracetamol, Simvastatin, Drugs for constipation, Cyanocobalamin, Mineral supplements, Amlodipine, Prednisolone, Opium derivatives and expectorants	1	*Hospital admission:* Fever, Type 2 diabetes, Unspecified tubal pregnancy *Drugs*: Furosemide, Paracetamol	1	*Hospital admission:* Urinary infection, Personal history of long-term (current) use of anticoagulants *Drugs*: Cyanocobalamin, Mineral supplements

Wave 1: 15 February 2020–26 June 2020; Wave 2: 05 November 2020–24 June 2021; Wave 3: 25 June 2021–31 December 2021. Hospital admission: diagnoses from both inpatient and outpatient specialist care. *Predictors type*.

**Table 3:** Candidate empirical covariates used for the development of the disease risk score for COVOID-19 mortality in Sweden.

Overall		Wave 1		Wave 2		Wave 3	
Weights	Predictors	Weights	Predictors	Weights	Predictors	Weights	Predictors
5	*Drugs*: Furosemide	5	*Drugs*: Furosemide	5	*Drugs*: Furosemide	5	
4	*Hospital admission:* Dyspnea, Unspecified tubal pregnancy, Unspecified atrial fibrillation and atrial flutter	4	*Hospital admission:* Unspecified tubal pregnancy, Dyspnea, Fever, Unspecified atrial fibrillation and atrial flutter *Drugs*: Paracetamol	4		4	*Hospital admission:* Unspecified tubal pregnancy *Drugs*: Furosemide
3	*Hospital admission:* Personal history of long-term (current) use of anticoagulants, Type 2 diabetes *Drugs*: Paracetamol, Drugs for constipation	3	*Hospital admission:* Type 2 diabetes, Personal history of long-term (current) use of anticoagulants, Urinary infection *Drugs*: Drugs for constipation	3	*Hospital admission:* Dyspnea, Unspecified atrial fibrillation and atrial flutter, Unspecified tubal, pregnancy *Drugs*: Paracetamol	3	*Hospital admission:* Unspecified atrial fibrillation and atrial flutter, Type 2 diabetes, Dyspnea, Personal, history of long-term (current) use of anticoagulants, Cataract, Nontraumatic compartment syndrome of left upper extremity *Drugs*: Paracetamol, Apixaban, Drugs for constipation
2	*Hospital admission:* Cataract, Fever, Urinary infection *Drugs*: Acetylsalicylic acid, Paracetamol, Metoprolol, Emollients and protectives, Cyanocobalamin, Mineral supplements	2	*Hospital admission:* Cataract *Drugs*: Acetylsalicylic acid, Metoprolol, Cyanocobalamin, Emollients and protectives, Mineral supplements, Paracetamol	2	*Hospital admission:* Personal history of long-term (current) use of anticoagulants, Type 2 diabetes *Drugs*: Paracetamol	2	*Drugs*: Metoprolol, Cyanocobalamin, Acetylsalicylic acid
1	*Hospital admission:* Age-related incipient cataract, Other retinal disorders, Presence of intraocular lens *Drugs*: Apixaban, Paracetamol, Oxycodone, Oxazepam, Omeprazole, Mineral supplements, Zopiclone, Bisoprolol, Cyanocobalamin, Metoprolol, Folic acid	1	*Drugs*: Apixaban		*Drugs*: Drugs for constipation	1	*Hospital admission:* Fever, Other specified disorders of skin and subcutaneous tissue, Age-related cataract, Presence of intraocular lens *Drugs*: Prednisolone, Emollients and protectives, Bisoprolol, Atorvastatin, Paracetamol, Oxycodone, Oxazepam, Simvastatin

Wave 1: 15 February 2020–26 June 2020; Wave 2: 05 November 2020–24 June 2021; Wave 3: 25 June 2021–31 December 2021. Wave 1: 15 February 2020–26 June 2020; Wave 2: 05 November 2020–24 June 2021; Wave 3: 25 June 2021–31 December 2021. Hospital admission: diagnoses from both inpatient and outpatient specialist care. *Predictors type*.

### 3.3 Disease risk score

The DRS included weights in a range between 1 and 5 (Tables 2, 3) which resulted in a DRS ranging between 0 and 8. The performance metrics for COVID 19-related hospitalization and mortality using the DRS are shown in Tables 4, 5 for Sweden and for the external validation in Norway, respectively. The density distribution of predicted probability of COVID-19 hospitalization and mortality including the density distribution of weights and prevalence in Sweden (overall and stratified by waves, age groups, and sex separately) are provided in Supplementary Figures 11–18.

**Table 4:** Models’ performance for COVID-19 hospitalization.

Wave	Stage	Model	AUC	Accuracy	Sensitivity	Specificity
Overall	Model development (Sweden)	*Logistic regression*	0.71	0.68	0.87	0.47
		*Ranger random forest*	0.71	0.68	0.86	0.50
		*Random partitioning*	0.64	0.66	0.90	0.34
		*Logistic regression (DRS)*	0.70	0.67	0.88	0.45
	External validation (Norway)	*Logistic regression (DRS)*	0.47	0.61	0.73	0.44
Wave 1	Model development (Sweden)	*Logistic regression*	0.77	0.74	0.88	0.58
		*Logistic regression using the DRS*	0.77	0.74	0.87	0.60
	External validation (Norway)	*Logistic regression (DRS)*	0.64	0.65	0.73	0.45
Wave 2	Model development (Sweden)	*Logistic regression*	0.73	0.69	0.85	0.51
		*Logistic regression using the DRS*	0.72	0.68	0.80	0.56
	External validation (Norway)	*Logistic regression (DRS)*	0.65	0.65	0.88	0.39
Wave 3	Model development (Sweden)	*Logistic regression*	0.68	0.65	0.84	0.45
		*Logistic regression using the DRS*	0.68	0.65	0.77	0.50
	External validation (Norway)	*Logistic regression (DRS)*	0.63	0.63	0.76	0.48

Disease Risk Score = DRS; Wave 1: 15 February 2020–26 June 2020; Wave 2: 05 November 2020–24 June 2021; Wave 3: 25 June 2021–31 December 2021.

**Table 5:** Models’ performance for COVID-19 mortality.

Wave	Stage	Model	AUC	Accuracy	Sensitivity	Specificity
Overall	Model development (Sweden)	*Logistic regression*	0.72	0.68	0.81	0.54
		*Logistic regression (DRS)*	0.72	0.68	0.75	0.60
	External validation (Norway)	*Logistic regression (DRS)*	0.74	0.73	0.67	0.73
Wave 1	Model development (Sweden)	*Logistic regression*	0.77	0.72	0.81	0.62
		*Logistic regression using the DRS*	0.76	0.72	0.75	0.67
	External validation (Norway)	*Logistic regression (DRS)*	0.58	0.65	0.81	0.33
Wave 2	Model development (Sweden)	*Logistic regression*	0.73	0.67	0.77	0.56
		*Logistic regression using the DRS*	0.71	0.67	0.72	0.59
	External validation (Norway)	*Logistic regression (DRS)*	0.70	0.72	0.60	0.74
Wave 3	Model development (Sweden)	*Logistic regression*	0.70	0.68	0.71	0.53
		*Logistic regression using the DRS*	0.66	0.67	0.60	0.61
	External validation (Norway)	*Logistic regression (DRS)*	0.75	0.73	0.61	0.74

Disease Risk Score = DRS; Wave 1: 15 February 2020–26 June 2020; Wave 2: 05 November 2020–24 June 2021; Wave 3: 25 June 2021–31 December 2021.

In the analysis stratified by age group and sex using the Swedish data, the density plots showed that the probability of hospitalization was positively correlated to DRS across the age groups and sex with some variation across the waves in the younger age groups (Supplementary Figures 11–14). Across all age groups and waves, the correlation between DRS and weights was consistently positive (*p* < 0.05) (Table 1 and Supplementary Table 2). When examining the relationship between DRS and prevalence, there was no correlation between the two variables among age groups and across waves (Supplementary Figures 11–14).

In all analyses, the peak of the density function reached the highest levels in the age groups above 65, suggesting higher median values of standardized DRS in these age groups when compared to the others (*p* < 0.05) (Supplementary Figures 11–14, Table 1, and Supplementary Table 2). This result was consistent in all single waves, too (Supplementary Figures 11–14, Table 1, and Supplementary Table 4). In wave 1 and 3, we observed higher median values of DRS among females while in wave 2 there was a slightly higher value of DRS among males (*p* < 0.05) (Supplementary Figures 11–14, Table 1, and Supplementary Table 4). No significant differences with respect to sex were observed across all waves (Supplementary Figures 15–18, Table 1, and Supplementary Table 4).

### 3.4 Models performance of the disease risk score and external validation

The performance of classification models is provided in Tables 4, 5. In the overall analysis for predicting COVID-19-related hospitalizations, various models were developed and externally validated. Logistic regression and Ranger random forest models had the best performance (Tables 4, 5). Logistic regression was prioritized over random forest due to its easily interpretable output.

#### 3.4.1 Hospitalization

The logistic regression model using the DRS had an AUC of 0.70, an accuracy of 0.67, a sensitivity of 0.88, and a specificity of 0.45 in Swedish data (Tables 4, 5). During external validation, the model had a performance of AUC 0.47, an accuracy of 0.61, a sensitivity of 0.73, and a specificity of 0.44. Similar performances were observed across waves (Tables 4, 5).

#### 3.4.2 Mortality

The logistic regression model using the DRS had an AUC of 0.72, an accuracy of 0.68, a sensitivity of 0.75, and a specificity of 0.60 in Swedish data (Tables 4, 5). During external validation, the model had a performance of AUC 0.74, an accuracy of 0.73, a sensitivity of 0.67, and a specificity of 0.73. Similar performances were observed across waves (Tables 4, 5).

#### 3.4.3 TRIPOD and PROBAST

The model’s performance was assessed using various evaluation metrics, including calibration and overall predictive accuracy, as recommended by the TRIPOD guidelines (Appendix 1). Overall, the model was classified as having a low risk of bias according to PROBAST (Appendix 2).

### 3.5 Formulas to calculate the probability of the outcome from the DRS

The formulas for the predicted probability of developing the outcome in the Swedish model for the overall period and separately by waves are provided in Table 6. These formulas were developed using the intercept and coefficients derived from the best classification model, specifically the logistic regression model. To express the mathematical representation of these formulas, we provide Formula 2 as follows:

**Table 6:** Formulas to predict the probability of COVID-19-related hospitalization and mortality in Sweden.

Period	Hospitalization	Mortality
Overall	Probabilityhosp.=1exp0.58−0.52∗DRS	Probabilitymort.=1exp0.77−0.30∗DRS
Wave 1	Probabilityhosp.=1exp0.88−0.60∗DRS	Probabilitymort.=1exp1.25−0.36∗DRS
Wave 2	Probabilityhosp.=1exp0.75−0.33∗DRS	Probabilitymort.=1exp0.89−0.27∗DRS
Wave 3	Probabilityhosp.=1exp0.52−0.32∗DRS	Probabilitymort.=1exp0.98−0.22∗DRS

DRS = Disease Risk Score; Wave 1: 15 February 2020–26 June 2020; Wave 2: 05 November 2020–24 June 2021; Wave 3: 25 June 2021–31 December 2021.

$\mathrm{Probability}\;\mathrm{of}\;\mathrm{the}\;\mathrm{outcome}=1/\left[\exp\left(\begin{array}{l} -\mathrm{intercept}\\ -{\mathrm{coefficient}}^{*}\;DRS \end{array}\right)\right]$

Formula 2. Formula to calculate the probability of the outcome using the DRS.

In Formula 2, “intercept” represents the intercept term obtained from the logistic regression model, “coefficient” refers to the respective coefficient associated with the DRS, and “DRS” represents the value of the DRS for a given individual. By substituting the appropriate values of DRS into this formula, it is possible to estimate the probability of the outcome.

For example, an individual who experienced severe dyspnea and high fever during the first wave of a COVID-19 infection, along with having type 2 diabetes as a risk factor, would be assigned a DRS of 12. This DRS indicates a high risk level and is associated with a 90% probability of hospitalization and subsequent mortality within 30 days following hospital admission.

## 4 Discussion

To our knowledge, this study represents the first register-based analysis utilizing high-quality Nordic data from Sweden and Norway to develop a comprehensive disease risk score for severe COVID-19 outcomes, including hospitalization and mortality.

### 4.1 Age and sex association with COVID-19 hospitalization and mortality

We observed a significantly higher risk of mortality and hospitalization for COVID-19 among men in both Sweden and Norway. This sex disparity aligns with previous research that has consistently reported a higher susceptibility and worse outcomes for males with COVID-19. The reasons behind this disparity may involve biological, behavioral, and social factors (39, 40). Additionally, our study found that younger patients (<65 years) had a lower risk of hospitalization and mortality for COVID-19. This finding is in line with previous studies (39, 40). The lower risk observed among younger individuals might be attributed to a more robust immune response or fewer underlying health conditions.

### 4.2 Predictors of COVID-19 hospitalization and mortality

It is not surprising to find dyspnea as one of the most important predictors. Dyspnea, or difficulty in breathing, is a common symptom associated with COVID-19 and is often linked to severe respiratory complications. It is a significant predictor of COVID-19 hospitalization, as individuals experiencing dyspnea may require specialized medical care to manage respiratory distress (41, 42).

High fever is another predictor that we found to be commonly associated with severe COVID-19, and its presence may indicate a more severe infection. While fever alone might not be sufficient to predict hospitalization, persistent or high-grade fevers can be indicative of systemic inflammation and severity of illness. In this regards, it is not surprising to find reimbursed prescriptions of paracetamol as a key predictor (43, 44).

We identified type 2 diabetes as a predictor for severe outcomes in COVID-19. Type 2 diabetes can contribute to an impaired immune response and increased vulnerability, which may necessitate hospitalization for appropriate clinical management (45–47). Individuals with type 2 diabetes are also more likely to have other comorbidities such as obesity, cardiovascular disease, and hypertension. Additionally, type 2 diabetes can cause damage to the blood vessels, leading to endothelial dysfunction. This impaired vascular function can contribute to the development of blood clots and other cardiovascular complications, which are seen in severe cases of COVID-19 (45–47). It is not surprising to find metformin among the predictors for COVID-19 hospitalization and mortality as this drug is often the first line treatment in type 2 diabetes.

Anticoagulant therapy was identified as a key predictor of COVID-19 hospitalization and mortality. The need for anticoagulants could reflect an underlying cardiovascular condition that increases the risk of severe COVID-19 and, consequently, the likelihood of hospitalization and mortality (48–50). We have identified atrial fibrillation as a predictors among cardiovascular conditions. Of note, amlodipine, enalapril, bisoprolol, apixaban, metoprolol, and simvastatin were identified as predictors for COVID 19-related hospitalization and mortality, serving as proxies of cardiovascular disorders. In this regard, it is important to emphasize that cardiovascular disorders previously have been identified as significant risk factors for COVID-19 hospitalization and mortality (48–50). Individuals with pre-existing conditions such as hypertension, coronary artery disease, congestive heart failure, and arrhythmias are more susceptible to severe outcomes. COVID-19 can exacerbate underlying cardiovascular issues, leading to increased risk of complications and poorer prognosis. The interaction between the virus and the cardiovascular system can cause inflammation, endothelial dysfunction, and thrombotic events (48–50).

While the direct relationship between cataracts and COVID-19 hospitalization is not clear, it is possible that older individuals with cataracts may have comorbidities or age-related vulnerabilities that contribute to a higher risk of hospitalization. A similar consideration applies to other ocular conditions such as the presence of intraocular lens and retinal disorders which, were identified as predictors in this study.

Urinary infections, or urinary tract infections (UTIs), are not directly caused by COVID-19 but can indirectly contribute to COVID-19 hospitalization and mortality. UTIs can lead to complications and worsen the health of individuals already susceptible to severe illness, such as older adults or those with underlying conditions. The presence of a UTI can trigger an immune response and systemic inflammation, potentially exacerbating the severity of COVID-19 (51, 52). While UTIs alone may not directly cause hospitalization or mortality in COVID-19 patients, their presence can indicate advanced age, underlying vulnerabilities and/or complications that may require hospitalization for specialized care (51, 52).

Oxycodone and other opioids are not directly linked to COVID-19 hospitalization or mortality. However, individuals prescribed these drugs may have underlying health conditions (e.g., cancer) or pain management needs that could influence COVID-19 outcomes. Factors such as underlying health conditions, respiratory depression caused by opioids, and potential immune system suppression may impact the severity of COVID-19 and increase the risk of hospitalization (53–55).

The use of drugs for constipation is not directly related to COVID-19 hospitalization or mortality. However, it can indirectly indicate underlying health conditions, potential polypharmacy and related adverse events, and general poor health status, which may influence COVID-19 outcomes. Underlying health conditions associated with chronic constipation could impact an individual’s overall health and immune system function, potentially increasing their vulnerability to severe COVID-19 outcomes. Polypharmacy and compromised gastrointestinal function could further complicate the health profile, increasing the risk of complications (56, 57).

Cyanocobalamin, also known as vitamin B12, is a micronutrient essential for various bodily functions, including red blood cell production and neurological health. While the direct relationship between cyanocobalamin and COVID-19 hospitalization and mortality is not clear, it plays a crucial role in maintaining overall health and immune function, and is often prescribed to older patients. Adequate levels of vitamin B12 are necessary for a robust immune response, and deficiencies in this vitamin may weaken the immune system’s ability to combat infections effectively. Consequently, individuals with low levels of cyanocobalamin may potentially be at a higher risk of severe COVID-19 outcomes, leading to an increased likelihood of hospitalization or mortality (58). The relationship between mineral supplements and COVID-19 hospitalization or mortality is not well-defined. While mineral supplements can contribute to overall health and immune function, their direct impact on COVID-19 outcomes is uncertain. Adequate mineral intake, including zinc, selenium, and vitamin D, is essential for a well-functioning immune system. However, the effectiveness of supplementation in preventing or treating COVID-19 is still under investigation. It is important to note that individual factors, such as baseline mineral levels, underlying health conditions, and dosage of supplements, can influence their impact (58–60).

Unspecified tubal pregnancy is not typically recognized as a direct risk factor for severe COVID-19 outcomes. Tubal pregnancy, also known as ectopic pregnancy, occurs when a fertilized egg implants outside of the uterus, usually in the fallopian tube. We therefore should consider these patients as hospitalized due to pregnancy outcomes who went through a COVID-19 screening. While ectopic pregnancy itself is not directly related to COVID-19 severity, pregnant individuals, in general, may be at a higher risk for severe outcomes if they contract the virus (61). Pregnancy is considered a risk factor for severe COVID-19 due to physiological changes that occur during gestation, including alterations in the immune system and respiratory function (61). Additionally, pregnant individuals may have an increased risk of complications due to the potential strain on the cardiovascular system (62, 63).

### 4.3 DRS performance

The density plots for the DRS indicate a positive correlation between the probability of hospitalization and the DRS across age groups and sexes. This means that as the DRS increases, indicating a higher risk score, the likelihood of hospitalization or death related to COVID-19 also increases. This finding suggests that the DRS is effective in predicting severe COVID-19 outcomes such as death across different demographic groups. However, it is worth noting that there is some variation of prediction performance of the DRS across the waves, particularly in the younger age groups. This variation may indicate changing patterns or factors influencing hospitalization risk in different time periods.

Regarding the analysis focusing on the correlation between the DRS and weights, it reveals a consistently positive correlation between the DRS and weights across all age groups and waves. The weights in the DRS reflect the importance or contribution of different risk factors in predicting hospitalization and death related to COVID-19. The positive correlation indicates that higher DRS values are associated with higher weights. In other words, risk factors with higher weights have a stronger influence on predicting hospitalization and death risks.

In the logistic regression model using the DRS as a predictor, the AUC was 0.70 in the Swedish data. The AUC is a measure of the model’s ability to distinguish between individuals who are hospitalized and those who are not. An AUC of 0.70 indicates a moderate level of accuracy. The model’s accuracy, which measures the overall correct prediction rate, was 0.67. This means that the model correctly predicted hospitalization status in 67% of cases. The sensitivity of the model, which measures the proportion of true positives identified, was 0.88 indicating that the model correctly identified 88% of individuals who were actually hospitalized. The specificity of the model, which measures the proportion of true negatives identified, was 0.45, meaning that the model correctly identified 45% of individuals who were not hospitalized.

During external validation, the performance of the model for predicting COVID-19 related hospitalization was low, with an AUC of 0.47, an accuracy of 0.61, a sensitivity of 0.73, and a specificity of 0.44. These results suggest that the model’s performance in predicting hospitalization was not as robust during external validation as it was within the Swedish data. Similar performances were observed across the different waves. Intuitively this might partly be attributed to loss of accuracy from using the section codes to build up the predictive model. We did not attempt to work the opposite way, to build up model with Norwegian data and validate with Swedish data. This was because the case numbers of individual waves in the Norwegian data was too small to have power at the first place. Additionally, this could also be due to differences in the healthcare systems in Sweden and Norway (e.g., thresholds for hospitalizations across waves). Additionally, significant differences in hospitalization criteria and the coding of ICD10 diagnoses may have been key factors in the observed results. These variations, influenced by differing reimbursement incentives, hindered the model’s effectiveness in a Norwegian context.

Regarding mortality, in the logistic regression model using the DRS as a predictor, the AUC for predicting mortality was 0.72 in the Swedish data. This indicates a moderately accurate model in distinguishing between individuals who died and those who survived. The accuracy of the model was 0.68, indicating an overall correct prediction rate of 68% for mortality. The sensitivity of the model was 0.75, indicating that it correctly identified 75% of individuals who actually died. The specificity of the model was 0.60, meaning it correctly identified 60% of individuals who did not die. During external validation, the model performed better, with an AUC of 0.74, an accuracy of 0.73, a sensitivity of 0.67, and a specificity of 0.73. These results indicate that the model’s performance in predicting mortality was relatively consistent across the Swedish data and the external validation set, with similar performances observed across waves.

In summary, the logistic regression model using the DRS showed moderate accuracy in predicting mortality but not hospitalization for COVID-19. The model had higher sensitivity, meaning it correctly identified a relatively high proportion of individuals who died. However, the specificity was lower, indicating a higher rate of false positives (individuals predicted to be hospitalized but who were not) when using hospitalization as an outcome.

Clinically, this score serves as a valuable tool for healthcare providers and researchers to gauge the potential risks associated with COVID-19. It enables a more precise identification of individuals who are at a higher risk of hospitalization and mortality, allowing for better resource allocation and patient management. However, it is crucial to recognize the inherent uncertainties in such risk prediction models. It is important to consider these performance metrics when interpreting and applying the DRS in clinical practice or public health decision-making, also taking into account that they may not be generalizable to other populations than the ones they were developed on and, eventually, for other time periods. By substituting the appropriate values of the DRS in the formulas provided in this article, one can estimate the probability of the outcome (i.e., death or hospitalization). In a clinical context, these formulas can be utilized to assess and predict the risk of COVID-19 hospitalization and mortality for individual patients. Clinicians can calculate the DRS for a patient based on their specific risk factors and then use formulas to estimate the probability of the outcome. This information can aid in clinical decision-making, such as determining the level of care likely to be needed, identifying high-risk individuals who may benefit from proactive interventions, and providing personalized recommendations for patients.

### 4.4 Strengths

One of the key strengths of our study is that we developed COVID-19 wave-specific models for hospitalization and mortality. Using a model developed to predict hospitalization and mortality for a wave of the COVID-19 pandemic for subsequent waves presents considerable challenges due to several critical factors. First and foremost, the virus itself has undergone significant evolution, giving rise to different variants with varying levels of aggressiveness. Notably, the Alpha variant demonstrated a heightened capacity to infect and impact the respiratory tract, thereby potentially leading to increased hospitalization and mortality rates compared to the earlier stages of the pandemic. Consequently, a model calibrated to the characteristics and dynamics of the first wave, where the original strain was predominant, may not adequately capture the distinct behaviors and outcomes associated with subsequent waves featuring novel variants.

Furthermore, it is crucial to consider the dynamic nature of the pandemic response. In the wake of the first wave, various countries and regions began implementing stringent public health contingency measures. The introduction of vaccines has had a profound impact on the epidemiological landscape, mitigating the severity of disease and reducing the strain on healthcare systems. Simultaneously, public health measures such as social distancing, mask mandates, and quarantine protocols have evolved in response to changing circumstances and scientific insights. These interventions, coupled with widespread vaccine distribution, have introduced new variables and altered the epidemiological dynamics, rendering a model developed for the first wave less applicable to subsequent waves.

In essence, the unique interplay of different virus variants, the evolving impact on the respiratory tract, and the introduction of vaccination and other contingency measures across various waves of the pandemic necessitate distinct models tailored to each specific wave. A model calibrated to the initial wave’s conditions and dynamics may not provide a comprehensive or accurate representation of the complex and evolving factors influencing hospitalization and mortality in later waves, making it crucial to adapt modeling approaches to the shifting landscape of the COVID-19 pandemic.

From a public health perspective, the formulas developed in our study can also be valuable for risk stratification at a population level. By applying the DRS to a larger population, public health officials can identify subgroups at higher risk of hospitalization or mortality. This information can guide resource allocation, public health interventions, and preventive measures, such as targeted vaccination campaigns or enhanced monitoring and support for high-risk individuals.

However, it is important to note that the predictive accuracy of these formulas should be considered in conjunction with other clinical information and the context in which they are applied. The performance metrics (AUC, accuracy, sensitivity, specificity) discussed earlier provide an assessment of the model’s overall predictive ability, but individual predictions may still have limitations and uncertainties, especially beyond the population that it was developed on. Therefore, these formulas should be interpreted and used as part of a comprehensive clinical assessment, considering other relevant factors such as patient history, comorbidities, and clinical judgment. Regular validation and refinement of the DRS and associated formulas based on real-world data are also essential to ensure their ongoing accuracy and reliability.

### 4.5 Limitations

This study’s results should considered in virtue of a set of strengths and Limitations. The study used linked data from national health registries in both Sweden and Norway, providing a robust and extensive dataset for analysis. This comprehensive data allowed for a thorough examination of various predictors and outcomes related to COVID-19 hospitalization and mortality. This large sample size enhances the statistical power and generalizability of the findings. The prescribed drug registers in both Sweden and Norway were used as proxies for drug information, and they are known to have complete coverage and high data quality. This strengthens the reliability and accuracy of the medication-related predictors included in the disease risk score (64).

However, a notable limitation is the absence of information on Over The Counter (OTC) drugs and diagnoses recorded in primary care within the dataset. This could lead to some misclassification and potential underestimation or incomplete representation of certain predictors (65). Additionally, primary care plays a significant role in healthcare consumption, particularly for chronic diseases and mental health conditions. The study may not fully capture the impact of these aspects due to the focus on in- and outpatient specialist care (65).

One potential limitation of our study is that we did not take into account ethnicity in Sweden when conducting the matching process between cases and controls, due to lack of such data in Sweden. Recent research has indicated that ethnicity can play a significant role in determining the severity of COVID-19 outcomes (66).

For future variants, the potential for utilizing our model under specific circumstances remains a possibility, contingent upon a couple of crucial factors. Firstly, there should be a substantial similarity in the pathophysiological aspects of the new COVID-19 variant with one of the previous strains. This is highly probable, given that the new variants detected thus far exhibit resemblances to those observed during the initial three waves of the pandemic. Secondly, an essential consideration is the presence of an epidemiological context akin to the one for which we have developed wave-specific models. In other words, the circumstances surrounding the spread, containment, and impact of the virus should align with those encountered during the waves for which our models were designed.

However, it is vital to also acknowledge that there will always be certain aspects that could significantly affect the validity and applicability of our models to future variants. These factors may include the emergence of entirely novel variants with distinct pathophysiological properties or epidemiological characteristics, or substantial changes in the public health and medical landscape, such as the introduction of new vaccines, treatments, or public health measures. Therefore, while our models provide a valuable framework, it’s imperative to approach each new variant with a degree of caution, recognizing that unforeseen variables can impact their predictive accuracy.

## 5 Conclusion

The DRS demonstrated moderate performance in predicting COVID-19-related mortality and poor performance for COVID-19-related hospitalization, with variations observed during external validation. Our study provides formulas to calculate the probability of the outcome using the DRS, which can be useful in clinical contexts for predicting individual risk and guiding public health interventions.

Overall, our study underscores the importance of proactive measures to prevent COVID-19 transmission, particularly among high-risk individuals. By prioritizing risk identification and implementing appropriate preventive strategies, we can strive to mitigate the impact of the pandemic on public health and improve patient outcomes.

## Data availability statement

The data analyzed in this study is subject to the following licenses/restrictions: available upon reasonable request to the authors. Requests to access these datasets should be directed to maurizio.sessa@sund.ku.dk.

## Ethics statement

Ethical approval was not required for the study involving humans in accordance with the local legislation and institutional requirements. Written informed consent to participate in this study was not required from the participants or the participants’ legal guardians/next of kin in accordance with the national legislation and the institutional requirements.

## Author contributions

SS: Conceptualization, Data curation, Formal analysis, Investigation, Methodology, Software, Writing – original draft, Writing – review & editing. JZ: Formal analysis, Methodology, Software, Writing – original draft, Writing – review & editing. HL: Data curation, Software, Writing – review & editing. HN: Conceptualization, Data curation, Funding acquisition, Resources, Writing – review & editing. AL: Conceptualization, Writing – review & editing. MP: Writing – review & editing. GS: Methodology, Writing – review & editing. FN: Funding acquisition, Methodology, Resources, Supervision, Writing – review & editing. BW: Conceptualization, Funding acquisition, Resources, Writing – review & editing. MH: Writing – review & editing. MA: Funding acquisition, Writing – review & editing. MS: Conceptualization, Data curation, Formal analysis, Investigation, Methodology, Software, Supervision, Validation, Visualization, Writing – original draft, Writing – review & editing.
