# Supplementary material for: Machine learning-driven development of a disease risk score for COVID-19 hospitalization and mortality: a Swedish and Norwegian register-based study
Source: Front Public Health. 2023 Dec 7;11:1258840. doi: 10.3389/fpubh.2023.1258840 (PMC10749372; doi:10.3389/fpubh.2023.1258840)
Supplement: Supplementary file 1 [file Data_Sheet_1.zip › Image 1.pdf]

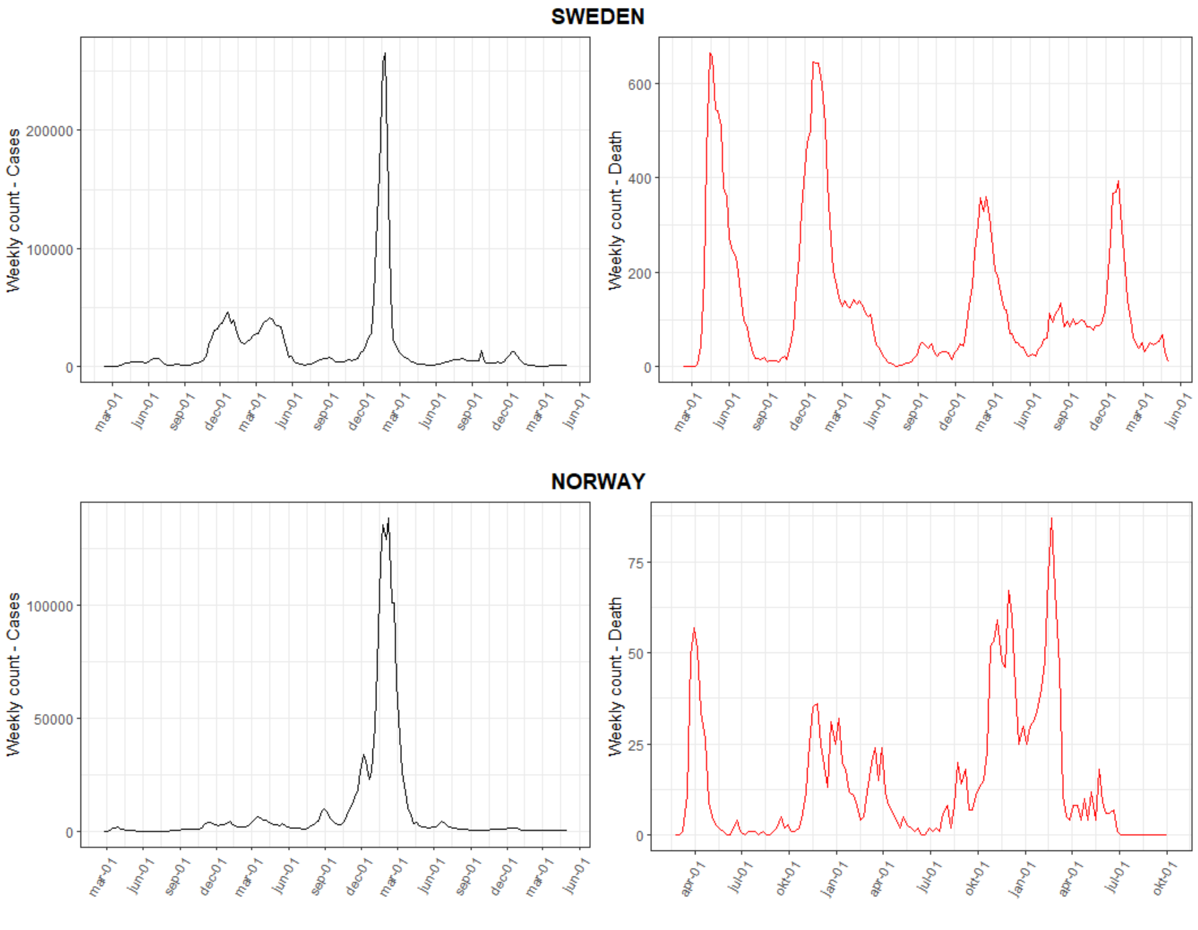

Supplementary Figure 1. Reported weekly counts of cases and deaths due to COVID-19 disease in Sweden and Norway from the 1st week of 2020 to the 18th week of 2023. *Data from: https://www.ecdc.europa.eu/en/publications-data/data-national-14-day-notification-rate-covid-19*
