# Supplementary material for: Machine learning-driven development of a disease risk score for COVID-19 hospitalization and mortality: a Swedish and Norwegian register-based study
Source: Front Public Health. 2023 Dec 7;11:1258840. doi: 10.3389/fpubh.2023.1258840 (PMC10749372; doi:10.3389/fpubh.2023.1258840)
Supplement: Supplementary file 1 [file Data_Sheet_1.zip › Image 2.pdf]

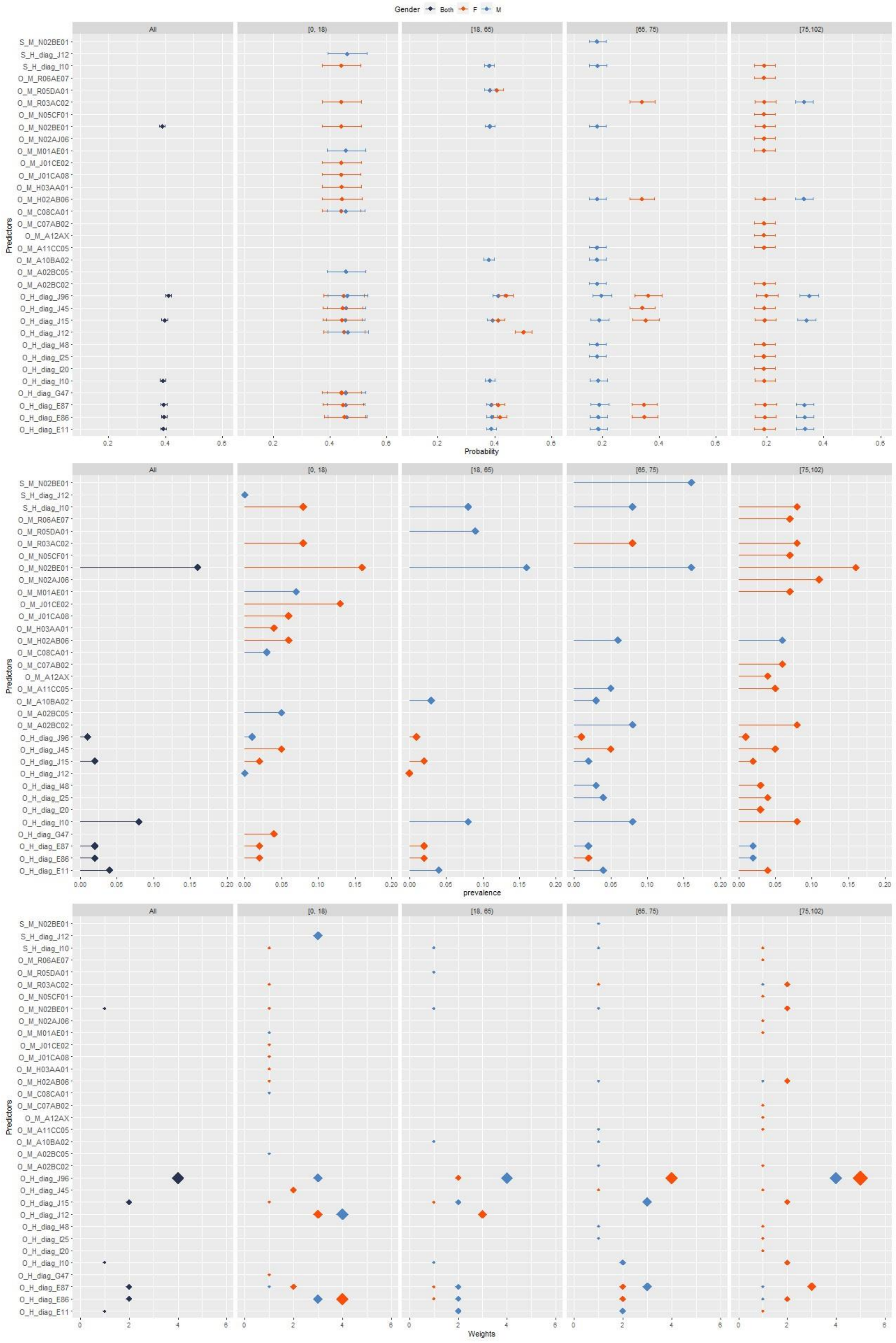

Supplementary Figure 2. *Norway:* stratified analysis by sex and age group for top-ranked predictors, their prevalence, weight, and predicted probability for COVID-19 hospitalization.
