# Supplementary material for: Machine learning-driven development of a disease risk score for COVID-19 hospitalization and mortality: a Swedish and Norwegian register-based study
Source: Front Public Health. 2023 Dec 7;11:1258840. doi: 10.3389/fpubh.2023.1258840 (PMC10749372; doi:10.3389/fpubh.2023.1258840)
Supplement: Supplementary file 1 [file Data_Sheet_1.zip › Table 2.docx]

**Supplementary table 2.** Granularity ATC codes in Sweden.

| **ATC codes** | **Code level** |
| --- | --- |
| A01 | Drug class |
| A02 | Drug class |
| A02BC | Full length |
| A02BD | Full length |
| A02BX | Full length |
| A03 | Drug class |
| A04 | Drug class |
| A05 | Drug class |
| A06 | Drug class |
| A07 | Drug class |
| A08 | Full length |
| A09 | Drug class |
| A10 | Full length |
| A11 | Drug class |
| A12 | Drug class |
| A13 | Drug class |
| A14 | Full length |
| A15 | Drug class |
| A16 | Drug class |
| B | Full length |
| C | Full length |
| D | Drug class |
| D11AH05 | Full length |
| G01 | Full length |
| G02 | Drug class |
| G03 | Full length |
| G04 | Full length |
| H | Full length |
| J | Full length |
| L | Full length |
| M | Full length |
| N | Full length |
| P01 | Full length |
| P02 | Drug class |
| P03 | Drug class |
| Q | One digit |
| R | Full length |
| S | Drug class |
| V | Drug class |
