# Supplementary material for: Machine learning-driven development of a disease risk score for COVID-19 hospitalization and mortality: a Swedish and Norwegian register-based study
Source: Front Public Health. 2023 Dec 7;11:1258840. doi: 10.3389/fpubh.2023.1258840 (PMC10749372; doi:10.3389/fpubh.2023.1258840)
Supplement: Supplementary file 1 [file Data_Sheet_1.zip › Table 3.docx]

**Supplementary Table 3.** Definitions of COVID-19 infection waves in Sweden and Norway, 2020-2021.

| **Wave** | **Wave period in Sweden** | **Wave period in Norway** |
| --- | --- | --- |
| 1 | 15 February 2020 - 26 June 2020 | 15 February 2020 - 04 June 2020 |
| 2 | 05 November 2020 – 24 June 2021 | 14 October 2020 – 05 June 2021 |
| 3 | 25 June 2021 - 31 December 2021 | 06 June 2021 -31 December 2021 |
