# Supplementary material for: Machine learning-driven development of a disease risk score for COVID-19 hospitalization and mortality: a Swedish and Norwegian register-based study
Source: Front Public Health. 2023 Dec 7;11:1258840. doi: 10.3389/fpubh.2023.1258840 (PMC10749372; doi:10.3389/fpubh.2023.1258840)
Supplement: Supplementary file 1 [file Data_Sheet_1.zip › Table 4.docx]

**Supplementary Table 4**. Demographic characteristics of cases and controls in Norway.

| **Hospitalization** | | | | | | **Mortality** | | | |
| --- | --- | --- | --- | --- | --- | --- | --- | --- | --- |
| ***Waves*** | ***Variable*** | ***Group*** | ***Control*** | ***Cases*** | ***Overall*** | ***Group*** | ***Control*** | ***Cases*** | ***Overall*** |
| **Overall**  **(%)** |  |  | ***N=42,909*** | ***N=10,835*** | ***N=53,744*** |  | ***N=3,467*** | ***N=928*** | ***N=4,395*** |
|  | *Age - group 1* | [0,18) | 1,904 (4.4) | 398  (3.7) | 2,302  (4.3) | [0,18) | 20  (0.6) | 4  (0.4) | 24  (0.5) |
|  | *Age - group 2* | [18,65) | 31,379 (73.1) | 7,067 (65.2) | 38,446 (71.5) | [18,65) | 759 (21.9) | 146 (15.7) | 905 (20.6) |
|  | *Age - group 3* | [65,75) | 4,917 (11.5) | 1,395 (12.9) | 6,312 (11.7) | [65,75) | 843 (24.3) | 173 (18.6) | 1,016 (23.1) |
|  | *Age - group 4* | [75,102] | 4,709 (11.0) | 1,975 (18.2) | 6,684 (12.4) | [75,102] | 1,845 (53.2) | 605 (65.2) | 2,450 (55.7) |
|  | *Sex* | M | 21,410 (49.9) | 5,640 (52.1) | 27,050 (50.3) | M | 2,099 (60.5) | 589 (63.5) | 2,688 (61.2) |
|  |  | F | 21,499 (50.1) | 5,195 (47.9) | 26,694 (49.7) | F | 1,368 (39.5) | 339 (36.5) | 1,707 (38.8) |
| **Wave 1**  **(%)** |  |  |  |  |  |  |  |  |  |
|  |  |  | ***N=5,262*** | ***N=1,666*** | ***N=6,928*** |  | ***N=587*** | ***N=152*** | ***N=739*** |
|  | *Age - group 1* | [0,18) | 65  (1.2) | 14  (0.8) | 79 (1.1) | [0,18) | 0  (0.0) | 0  (0.0) | 0  (0.0) |
|  | *Age - group 2* | [18,65) | 3,862 (73.4) | 974  (58.5) | 4,836 (69.8) | [18,65) | 122 (20.8) | 22 (14.5) | 144 (19.5) |
|  | *Age - group 3* | [65,75) | 711 (13.5) | 269  (16.1) | 980 (14.1) | [65,75) | 139 (23.7) | 25 (16.4) | 164 (22.2) |
|  | *Age - group 4* | [75,102] | 624 (11.9) | 409  (24.5) | 1,033 (14.9) | [75,102] | 326 (55.5) | 105 (69.1) | 431 (58.3) |
|  | *Sex* | M | 2,716 (51.6) | 910  (54.6) | 3,626 (52.3) | M | 334 (56.9) | 97 (63.8) | 431 (58.3) |
|  |  | F | 2,546 (48.4) | 756  (45.4) | 3,302 (47.7) | F | 253 (43.1) | 55 (36.2) | 308 (41.7) |
|  |  |  |  |  |  |  |  |  |  |
| **Wave 2**  **(%)** |  |  | ***N=18,292*** | ***N=4,756*** | ***N=23,048*** |  | ***N=1,475*** | ***N=367*** | ***N=1,842*** |
|  | *Age - group 1* | [0,18) | 550 (3.0) | 123  (2.6) | 673  (2.9) | [0,18) | 15  (1.0) | 3 (0.8) | 18 (1.0) |
|  | *Age - group 2* | [18,65) | 14,015 (76.6) | 3,230 (67.9) | 17,245 (74.8) | [18,65) | 389 (26.4) | 74 (20.2) | 463 (25.1) |
|  | *Age - group 3* | [65,75) | 2,224 (12.2) | 649  (13.6) | 2,873 (12.5) | [65,75) | 422 (28.6) | 88 (24.0) | 510 (27.7) |
|  | *Age - group 4* | [75,102] | 1,503 (8.2) | 754  (15.9) | 2,257 (9.8) | [75,102] | 649 (44.0) | 202 (55.0) | 851 (46.2) |
|  | *Sex* | M | 9,580 (52.4) | 2,604 (54.8) | 12,184 (52.9) | M | 904 (61.3) | 224 (61.0) | 1,128 (61.2) |
|  |  | F | 8,712 (47.6) | 2,152 (45.2) | 10,864 (47.1) | F | 571 (38.7) | 143 (39.0) | 714 (38.8) |
|  |  |  |  |  |  |  |  |  |  |
| **Wave 3**  **(%)** |  |  | ***N=18,083*** | ***N=4,040*** | ***N=22,123*** |  | ***N=1,593*** | ***N=393*** | ***N=1,986*** |
|  | *Age - group 1* | [0,18) | 1,236 (6.8) | 250 (6.2) | 1,486 (6.7) | [0,18) | 5 (0.3) | 1 (0.3) | 6 (0.3) |
|  | *Age - group e 2* | [18,65) | 12,520 (69.2) | 2,624 (65.0) | 15,144 (68.5) | [18,65) | 242 (15.2) | 47 (12.0) | 289 (14.6) |
|  | *Age - group 3* | [65,75) | 1,858 (10.3) | 434 (10.7) | 2,292 (10.4) | [65,75) | 289 (18.1) | 58 (14.8) | 347 (17.5) |
|  | *Age - group 4* | [75,102] | 2,469 (13.7) | 732 (18.1) | 3,201 (14.5) | [75,102] | 1,057 (66.4) | 287 (73.0) | 1,344 (67.7) |
|  | *Sex* | M | 8,473 (46.9) | 1,928 (47.7) | 10,401 (47.0) | M | 1,036 (65.0) | 259 (65.9) | 1,295 (65.2) |
|  |  | F | 9,610 (53.1) | 2,112 (52.3) | 11,722 (53.0) | F | 557 (35.0) | 134 (34.1) | 691 (34.8) |
