# Supplementary material for: Machine learning-driven development of a disease risk score for COVID-19 hospitalization and mortality: a Swedish and Norwegian register-based study
Source: Front Public Health. 2023 Dec 7;11:1258840. doi: 10.3389/fpubh.2023.1258840 (PMC10749372; doi:10.3389/fpubh.2023.1258840)
Supplement: Supplementary file 1 [file Data_Sheet_1.zip › Table 5.docx]

**Supplementary table 5.** Overview of candidate empirical covariates prioritization procedures.

| **Waves** | **Outcome** | **Number of candidate empirical covariates** | **Candidate empirical covariates after filtering by variance** | **Prioritized candidate empirical covariates used in the disease risk score** |
| --- | --- | --- | --- | --- |
| Overall | Hospitalization | 23,607 | 69 | 6 |
|  | Mortality | 23,607 | 69 | 31 |
| Wave 1 | Hospitalization | 23,607 | 69 | 34 |
|  | Mortality | 23,607 | 69 | 18 |
| Wave 2 | Hospitalization | 23,607 | 69 | 6 |
|  | Mortality | 23,607 | 69 | 9 |
| Wave 3 | Hospitalization | 23,607 | 69 | 14 |
|  | Mortality | 23,607 | 69 | 26 |
