# Supplementary material for: Machine learning-driven development of a disease risk score for COVID-19 hospitalization and mortality: a Swedish and Norwegian register-based study
Source: Front Public Health. 2023 Dec 7;11:1258840. doi: 10.3389/fpubh.2023.1258840 (PMC10749372; doi:10.3389/fpubh.2023.1258840)
Supplement: Supplementary file 1 [file Data_Sheet_1.zip › Table 1.docx]

**Supplementary table 1.** Granularity ICD-10 codes in Sweden.

| **ICD-10 codes** | **Code level** |
| --- | --- |
| A00-A99 | Full length |
| B00-B99 | Full length |
| C00-C39 | Full length |
| C40-C97 | 3-level |
| D00-D77 | 3-level |
| D693 D694 D695 | Full length |
| D80-D89 | Full length |
| E00-E35 | Full length |
| E40-E64 | Section |
| E65-E68 | Full length |
| E70-E90 | 3-level |
| F00-F09 | Full length |
| F10-F99 | 3-level |
| G00-G09 | 3-level |
| G030 | Full length |
| G10-G26 | 3-level |
| G114 | Full length |
| G30-G47 | 3-level |
| G311 G474 | Full length |
| G50-G73 | 3-level |
| G510 G610 G700 | Full length |
| G80-G83 | 3-level |
| G801 G802 G830 G831 G832 G833 G834 G839 | Full length |
| G90-G99 | Section |
| H00-H99 | 3-level |
| H340 | Full length |
| I00-I99 | Full length |
| J00-J99 | Full length |
| K00-K77 | Full length |
| K80-K93 | Section |
| K85 | Full length |
| L00-L54 | Full length |
| L55-L99 | Section |
| L95 | 3-level |
| M00-M14 | Full length |
| M15-M25 | 3-level |
| M255 | Full length |
| M30-M36 | Full length |
| M40-M99 | Section |
| N00-N19 | Full length |
| N20-N99 | Section |
| O00-O99 | 3-level |
| O119 O244 O601 O603 O904 | Full length |
| P00-P99 | 3-level |
| P072 P073 P101 P220 P290 P524 P526 P610 | Full length |
| Q00-Q99 | Section |
| R00-R69 | Full length |
| R70-R99 | Section |
| R96 | 3-level |
| S00-S99 | Section |
| T00-T99 | 3-level |
| T691 T782 T805 T880 T881 T886 | Full length |
| U00-U49 | Full length |
| U82-U85 | Section |
| U98-U99 | Full length |
| V00-V99 | Section |
| W00-W99 | Section |
| X00-X99 | Section |
| Y00-Y99 | 3-level |
| Y590 | Full length |
| Z00-Z99 | Full length |
