# Supplementary material for: Machine learning-driven development of a disease risk score for COVID-19 hospitalization and mortality: a Swedish and Norwegian register-based study
Source: Front Public Health. 2023 Dec 7;11:1258840. doi: 10.3389/fpubh.2023.1258840 (PMC10749372; doi:10.3389/fpubh.2023.1258840)
Supplement: Supplementary file 1 [file Data_Sheet_1.zip › PROBAST.docx]

## PROBAST

(Prediction model study Risk Of Bias Assessment Tool)

Published in Annals of Internal Medicine (freely available):

1. [PROBAST: A Tool to Assess the Risk of Bias and Applicability of Prediction Model Studies](https://annals.org/aim/fullarticle/2719961/probast-tool-assess-risk-bias-applicability-prediction-model-studies)
2. [PROBAST: A Tool to Assess Risk of Bias and Applicability of Prediction Model Studies: Explanation and Elaboration](https://annals.org/aim/fullarticle/2719962/probast-tool-assess-risk-bias-applicability-prediction-model-studies-explanation)

| **What does PROBAST assess?**  PROBAST assesses both the *risk of bias* and *concerns regarding applicability* of a study that evaluates (develops, validates or updates) a multivariable diagnostic or prognostic prediction model. It is designed to assess primary studies included in a systematic review.  *Bias* occurs if systematic flaws or limitations in the design, conduct or analysis of a primary study distort the results. For the purpose of prediction modelling studies, we have defined *risk of bias* to occur when shortcomings in the study design, conduct or analysis lead to systematically distorted estimates of a model’s predictive performance or to an inadequate model to address the research question. Model predictive performance is typically evaluated using calibration, discrimination and sometimes classification measures, and these are likely inaccurately estimated in studies with high risk of bias. *Applicability* refers to the extent to which the prediction model from the primary study matches your systematic review question, for example in terms of the participants, predictors or outcome of interest.  A primary study may include the development and/or validation or update of more than one prediction model. A PROBAST assessment should be completed for each distinct model that is developed, validated or updated (extended) for making individualised predictions. Where a publication assesses multiple prediction models, only complete a PROBAST assessment for those models that meet the inclusion criteria for your systematic review. Please note that subsequent use of the term “model” includes derivatives of models, such as simplified risk scores, nomograms, or recalibrations of models.  PROBAST is not designed for all multivariable diagnostic or prognostic studies. For example, studies using multivariable models to identify predictors associated with an outcome but not attempting to develop a model for making individualised predictions are not covered by PROBAST.  PROBAST includes four steps.   \| **Step** \| **Task** \| **When to complete** \| \| --- \| --- \| --- \| \| **1** \| Specify your systematic review question(s) \| Once per systematic review \| \| **2** \| Classify the type of prediction model evaluation \| Once for each model of interest in each publication being assessed, for each relevant outcome \| \| **3** \| Assess risk of bias and applicability \| Once for each development and validation of each distinct prediction model in a publication \| \| **4** \| Overall judgment \| Once for each development and validation of each distinct prediction model in a publication \|   If this is your first time using PROBAST, we strongly recommend reading the detailed explanation and elaboration (E&E, see link above) paper and to check the examples on www.probast.org |
| --- | --- | --- | --- | --- | --- | --- | --- | --- | --- | --- | --- | --- | --- | --- | --- |

**Step 1: Specify your systematic review question**

| State your systematic review question to facilitate the assessment of the applicability of the evaluated models to your question. *The following table should be completed once per systematic review.* |
| --- |

| **Criteria** | **Specify your systematic review question** |
| --- | --- |
| *Intended use of model:* | To develop a disease risk score for COVID-19-related hospitalization and mortality in Sweden and externally validate it in Norway |
| ***Participants*** *including selection criteria and setting:* | 124,560 residents in Sweden with SARS-CoV-2 infection confirmed by RT-PCR test up to August 2022 and 53,744 residents in Norway with COVID-19 up to December 2021 were selected as study cohort. Diagnoses for in- and outpatient specialist care, prescribed drug dispensing and deaths were used. |
| ***Predictors*** *(used in prediction modelling), including types of predictors (e.g. history, clinical examination, biochemical markers, imaging tests), time of measurement, specific measurement issues (e.g., any requirements/ prohibitions for specialized equipment):* | Demographics, infectious, somatic, and mental health diseases, recorded diagnoses, and pharmacological treatments. |
| *Outcome to be predicted:* | COVID-19-related hospitalization and mortality |

**Step 2: Classify the type of prediction model evaluation**

| Use the following table to classify the evaluation as model development, model validation or model update, or combination. Different signalling questions apply for different types of prediction model evaluation. If the evaluation does not fit one of these classifications then PROBAST should not be used. |
| --- |

| **Classify the evaluation based on its aim** | | | |
| --- | --- | --- | --- |
| **Type of prediction study** | **PROBAST boxes to complete** | **Tick as appropriate** | **Definition for type of prediction model study** |
| Development only | Development | ✖ | Prediction model development without external validation. These studies may include internal validation methods, such as bootstrapping and cross-validation techniques. |
| Development and validation | Development and validation | ✓ | Prediction model development combined with external validation in other participants in the same article. |
| Validation only | Validation | ✖ | External validation of existing (previously developed) model in other participants. |

| *This table should be completed once for each publication being assessed and for each relevant outcome in your review.* | |  |
| --- | --- | --- |
| **Publication reference** |  | |
| **Models of interest** | random partitioning, ranger random forest, logistic regression and disease risk score development | |
| **Outcome of interest** | COVID-19 hospitalization and death | |

**Step 3: Assess risk of bias and applicability**

| PROBAST is structured as four key domains. Each domain is judged for risk of bias (low, high or unclear) and includes signalling questions to help make judgements. Signalling questions are rated as yes (Y), probably yes (PY), probably no (PN), no (N) or no information (NI). All signalling questions are phrased so that “yes” indicates absence of bias. Any signalling question rated as “no” or “probably no” flags the potential for bias; you will need to use your judgement to determine whether the domain should be rated as “high”, “low” or “unclear” risk of bias. The guidance document contains further instructions and examples on rating signalling questions and risk of bias for each domain.  The first three domains are also rated for concerns regarding applicability (low/ high/ unclear) to your review question defined above.  *Complete all domains separately for each evaluation of a distinct model. Shaded boxes indicate where signalling questions do not apply and should not be answered.* |
| --- |

| **DOMAIN 1: Participants** | | | |
| --- | --- | --- | --- |
| **A. Risk of Bias** | | | |
| *Describe the sources of data and criteria for participant selection:*  Dev: The source populations of all residents (2.6 million) in Sweden with COVID-19 were used. Then  (n=124,560) were selected from November 2019 to August 2022 who were admitted to hospital for COVID-19 (International Classification of Diseases version 10, *ICD-10:* U07) up to 14 days after the positive test (i.e., cases). The Swedish data originated from the SCIFI-PEARL (Swedish COVID-19 Investigation for Future Insights – a Population The national database of notifiable diseases (Sminet) was used to identify positive SARS-CoV-2 RT-PCR test results.  Val: The source populations of patients with COVID-19 were 0.4 million in Norway. Then 53,744 residents in Norway from February 2020 to December 2021 who were hospitalized for COVID_19 up to 14 days after the positive test were selected. The Norwegian Surveillance System for Communicable Diseases (MSIS) was used to obtain information on notified infectious diseases including SARS-Cov-2, and the Norwegian Patient Registry (NPR) to identify individuals hospitalized for COVID-19. | | | |
|  | | Dev | Val |
| - 1. Were appropriate data sources used, e.g. cohort, RCT or nested case-control study data? | | Y | Y |
| - 1. Were all inclusions and exclusions of participants appropriate? | | Y | Y |
| **Risk of bias introduced by selection of participants** | **RISK:**  *(low/ high/ unclear)* | **low** | **low** |
| *Rationale of bias rating:* | | | |
| This data is part of the general population. | | | |
| **B. Applicability** | | | |
| *Describe included participants, setting and dates:*  The setting described in above were also performed separately within data from the COVID-19 waves. | | | |
| **Concern that the included participants and setting do not match the review question** | **CONCERN:**  *(low/ high/ unclear)* | **low** | **low** |
| *Rationale of applicability rating:* No Concerns | | | |
|  | | | |

| **DOMAIN 2: Predictors** | | | |
| --- | --- | --- | --- |
| **A. Risk of Bias** | | | |
| *List and describe predictors included in the final model, e.g. definition and timing of assessment:*  dyspnea, fever, chronic conditions such as type 2 diabetes and atrial fibrillation and several drugs such as metformin, enalapril, amlodipine, bisoprolol, metoprolol, prednisolone and bronchodilators.  To identify dispensed prescriptions for medicine in Sweden and Norway using a covariate assessment window of 365 days before the index date, while for diagnoses and surgery/procedures from hospital inpatient admissions and specialist outpatient visits, they used all the information available in Sweden and Norway before the index date. | | | |
|  | | Dev | Val |
| - 1. Were predictors defined and assessed in a similar way for all participants? | | Y | Y |
| - 1. Were predictor assessments made without knowledge of outcome data? | | Y | Y |
| - 1. Are all predictors available at the time the model is intended to be used? | | Y | Y |
| **Risk of bias introduced by predictors or their assessment** | **RISK:**  *(low/ high/ unclear)* | **low** | **low** |
| *Rationale of bias rating:*  No Concerns | | | |
| **B. Applicability** | | | |
| Concern that the definition, assessment or timing of predictors in the model do not match the review question | **CONCERN:**  *(low/ high/ unclear)* | **low** | **low** |
| *Rationale of applicability rating:*  Predictors were assessed prior to the occurrence of the outcome. Therefore, all predictors included in this article can be considered valid.  They used two covariant assessment windows to capture the predictors which is clearly defined. | | | |

| **DOMAIN 3: Outcome** | | | |
| --- | --- | --- | --- |
| **A. Risk of Bias** | | | |
| *Describe the outcome, how it was defined and determined, and the time interval between predictor assessment and outcome determination:*  Hospitalized cases were defined as hospitalization within 14 days after a positive SARS-CoV-2 test.  Individuals who died within 30 days after COVID-19-related hospitalization were identified as COVID-19 death cases. | | | |
|  | | Dev | Val |
| - 1. Was the outcome determined appropriately? | | Y | Y |
| - 1. Was a pre-specified or standard outcome definition used? | | Y | Y |
| - 1. Were predictors excluded from the outcome definition? | | NI | NI |
| - 1. Was the outcome defined and determined in a similar way for all participants? | | Y | Y |
| - 1. Was the outcome determined without knowledge of predictor information? | | Y | Y |
| - 1. Was the time interval between predictor assessment and outcome determination appropriate? | | Y | Y |
| **Risk of bias introduced by the outcome or its determination** | **RISK:**  *(low/ high/ unclear)* | **low** | **low** |
| *Rationale of bias rating:*  No Concerns. | | | |
| **B. Applicability** | | | |
| *At what time point was the outcome determined:*  The study has an advantage of using different time period for Covid-19 waves.  *If a composite outcome was used, describe the relative frequency/distribution of each contributing outcome:* | | | |
| **Concern that the outcome, its definition, timing or determination do not match the review question** | **CONCERN:**  *(low/ high/ unclear)* | **low** | **low** |
| *Rationale of applicability rating:*  The study has an advantage of developing models based on differentCovid-19 waves and external evaluating on a large population. | | | |

| **DOMAIN 4: Analysis** | | | |
| --- | --- | --- | --- |
| **Risk of Bias** | | | |
| *Describe numbers of participants, number of candidate predictors, outcome events and events per candidate predictor:*  Dev: 124,560 (23.1%) out of 538,277 Swedish individuals were hospitalized and 15,877 (2.9%) died within 30 days following COVID-19 hospitalization during the study periods.  In total, there were 23,607 candidate empirical covariates generated during the data management phase for Swedish data sources , and the number of variables after filtering by variance was reduced to 69.  Val: 10,835(20.2%) out of 53,744 Norwegian hospitalized for COVID-19 and 928 (1.7%) died within 30 days following COVID-19 hospitalization. | | | |
| *Describe how the model was developed (for example in regards to modelling technique (e.g. survival or logistic modelling), predictor selection, and risk group definition):*  The Disease Risk Score was developed included selected predictors and their weights in a range between 1 and 5 which resulted in a DRS ranging between 0 and 8.  The normalized ensemble score of the 6 prioritization methods was used for ranking features’ importance which was then used to identify the optimal number of features. The optimal number of features was identified by looking at the deviation of the AUC. | | | |
| *Describe whether and how the model was validated, either internally (e.g. bootstrapping, cross validation, random split sample) or externally (e.g. temporal validation, geographical validation, different setting, different type of participants):*  A 5-fold cross-validation method was applied. Overall model performance was assessed by averaging model performances for each fold. For assessing the model performance, the accuracy, AUC, sensitivity, specificity, and accuracy were measured for all models using a confusion matrix.  The DRSs developed using the Swedish data across multiple waves of data collection were externally validated in Norway. | | | |
| *Describe the performance measures of the model, e.g. (re)calibration, discrimination, (re)classification, net benefit, and whether they were adjusted for optimism:*  Calibration was performed to get bias-corrected (overfitting-corrected) estimates of predicted probabilities using the DRS. They have plotted the calibrated predicted probability of developing the outcome given the standardized DRS, the weights and the prevalence of each predictor and they provided the formula to obtain the probability of the outcome given the value of the DRS. | | | |
| *Describe any participants who were excluded from the analysis:*  Not available. | | | |
| *Describe missing data on predictors and outcomes as well as methods used for missing data:*  Not available | | | |
|  | | Dev | Val |
| - 1. Were there a reasonable number of participants with the outcome? | | Y | Y |
| - 1. Were continuous and categorical predictors handled appropriately? | | Y | Y |
| - 1. Were all enrolled participants included in the analysis? | | Y | Y |
| - 1. Were participants with missing data handled appropriately? | | NI | NI |
| - 1. Was selection of predictors based on univariable analysis avoided? | | NI |  |
| - 1. Were complexities in the data (e.g. censoring, competing risks, sampling of controls) accounted for appropriately? | | Y | Y |
| - 1. Were relevant model performance measures evaluated appropriately? | | Y | Y |
| - 1. Were model overfitting and optimism in model performance accounted for? | | Y |  |
| - 1. Do predictors and their assigned weights in the final model correspond to the results from multivariable analysis? | | Y |  |
| **Risk of bias introduced by the analysis** | **RISK:**  *(low/ high/ unclear)* | **low** | **low** |
| *Rationale of bias rating:*  The analysis benefited from a large number of participants both for development and validation. | | | |

**Step 4: Overall assessment**

| Use the following tables to reach overall judgements about risk of bias and concerns regarding applicability of the prediction model evaluation (development and/or validation) across all assessed domains.  *Complete for each evaluation of a distinct model.*   \| **Reaching an overall judgement about risk of bias of the prediction model evaluation** \| \| \| --- \| --- \| \| **Low risk of bias** \| If all domains were rated low risk of bias.  If a prediction model was developed without any external validation, and it was rated as low risk of bias for all domains, consider downgrading to **high risk of bias**. Such a model can only be considered as low risk of bias, if the development was based on a very large data set and included some form of internal validation. \| \| **High risk of bias** \| If at least one domain is judged to be at **high risk of bias**. \| \| **Unclear risk of bias** \| If an unclear risk of bias was noted in at least one domain and it was low risk for all other domains. \|  \| **Reaching an overall judgement about applicability of the prediction model evaluation** \| \| \| --- \| --- \| \| **Low concerns regarding applicability** \| If low concerns regarding applicability for all domains, the prediction model evaluation is judged to have **low concerns regarding applicability**. \| \| **High concerns regarding applicability** \| If high concerns regarding applicability for at least one domain, the prediction model evaluation is judged to have **high concerns regarding applicability**. \| \| **Unclear concerns regarding applicability** \| If unclear concerns (but no “high concern”) regarding applicability for at least one domain, the prediction model evaluation is judged to have **unclear concerns regarding applicability** overall. \| |
| --- | --- | --- | --- | --- | --- | --- | --- | --- | --- | --- | --- | --- | --- | --- | --- | --- |

| **Overall judgement about risk of bias and applicability of the prediction model evaluation** | | |
| --- | --- | --- |
| **Overall judgement of risk of bias** | **RISK:**  *(low/ high/ unclear)* | **low** |
| *Summary of sources of potential bias:*  The study has been done from the different pandemic waves. Also the analysis benefited from a large number of participants both for development and validation. | | |
| **Overall judgement of applicability** | **CONCERN:**  *(low/ high/ unclear)* | **low** |
| *Summary of applicability concerns:*  No Concern. | | |
