# Supplementary figures and images for: Machine learning-driven development of a disease risk score for COVID-19 hospitalization and mortality: a Swedish and Norwegian register-based study
Source: Front Public Health. 2023 Dec 7;11:1258840. doi: 10.3389/fpubh.2023.1258840 (PMC10749372; doi:10.3389/fpubh.2023.1258840)

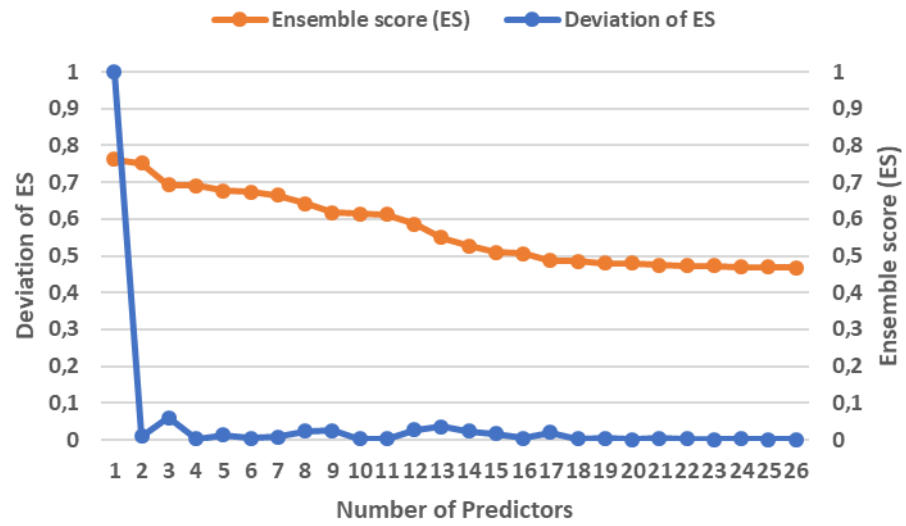

**Supplementary figure 10.** Deviation of the ensemble score – Wave 3 period – Mortality.

Supplement: Supplementary file 1 [file Data_Sheet_1.zip › Image 10.pdf]

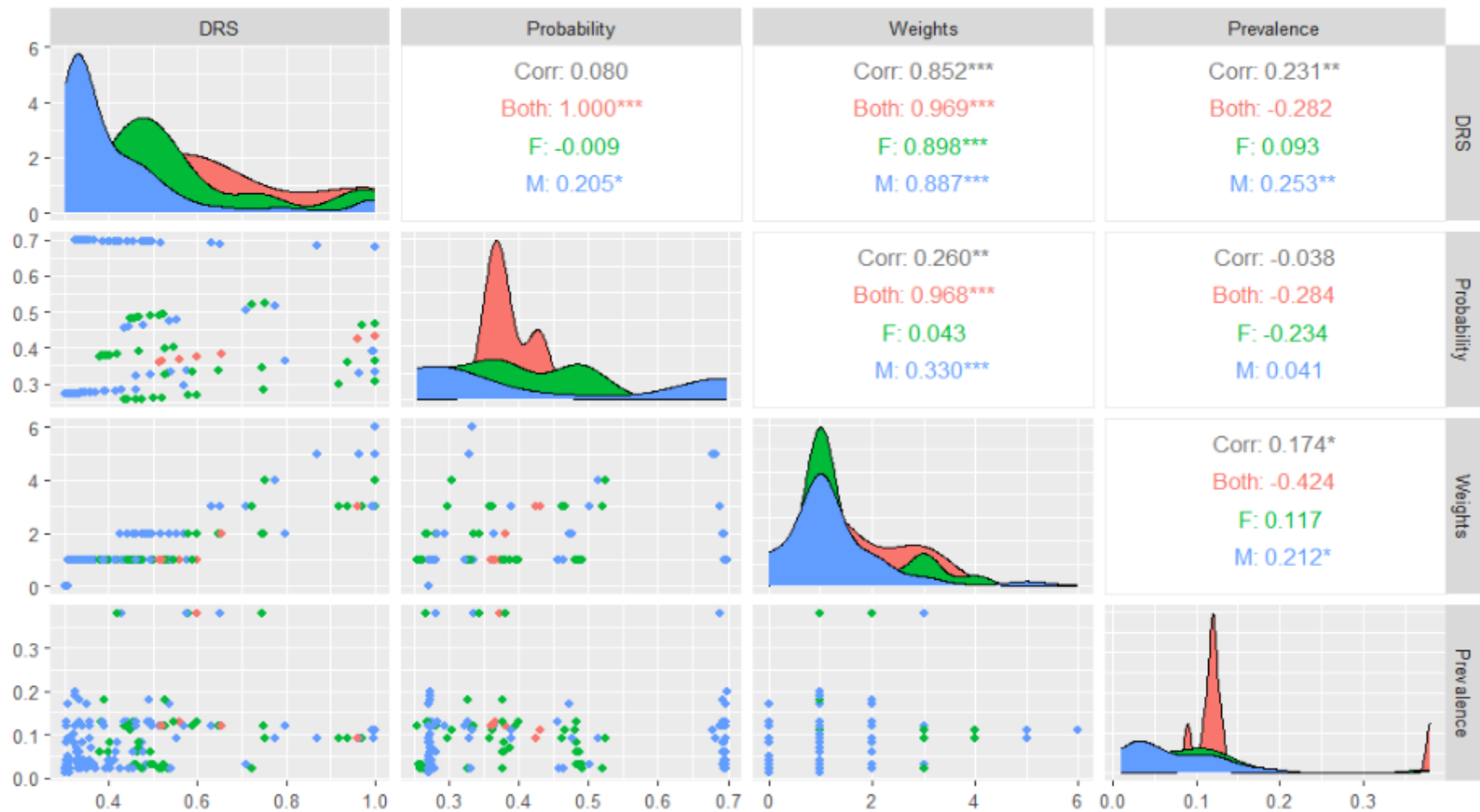

**Supplementary figure 16.** Pairwise correlation plots by sex – Wave 1, Sweden. *Disease Risk Score = DRS*

Supplement: Supplementary file 1 [file Data_Sheet_1.zip › Image 16.pdf]

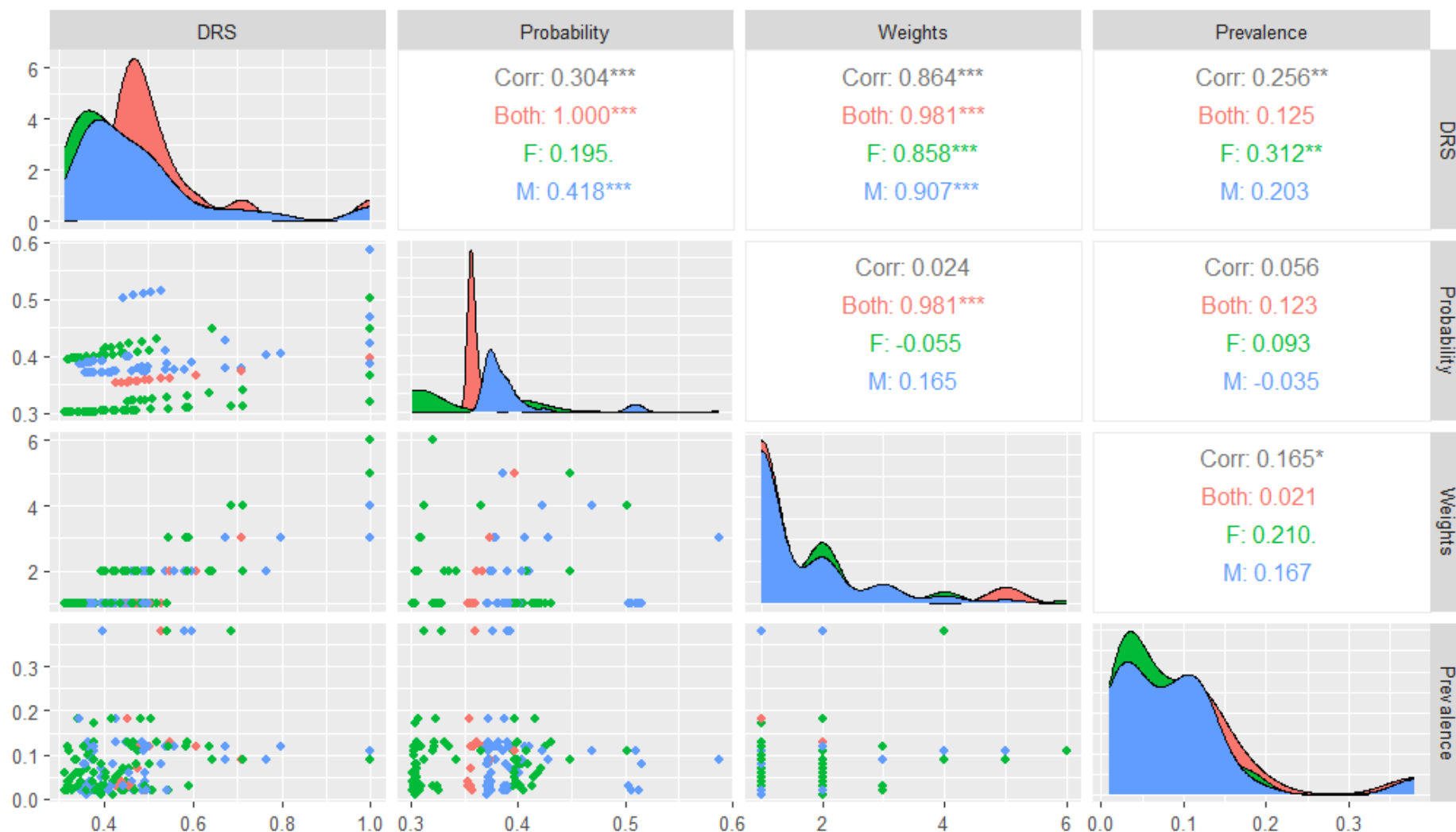

**Supplementary figure 17.** Pairwise correlation plots by sex – Wave 2, Sweden. *Disease Risk Score = DRS*

Supplement: Supplementary file 1 [file Data_Sheet_1.zip › Image 17.pdf]

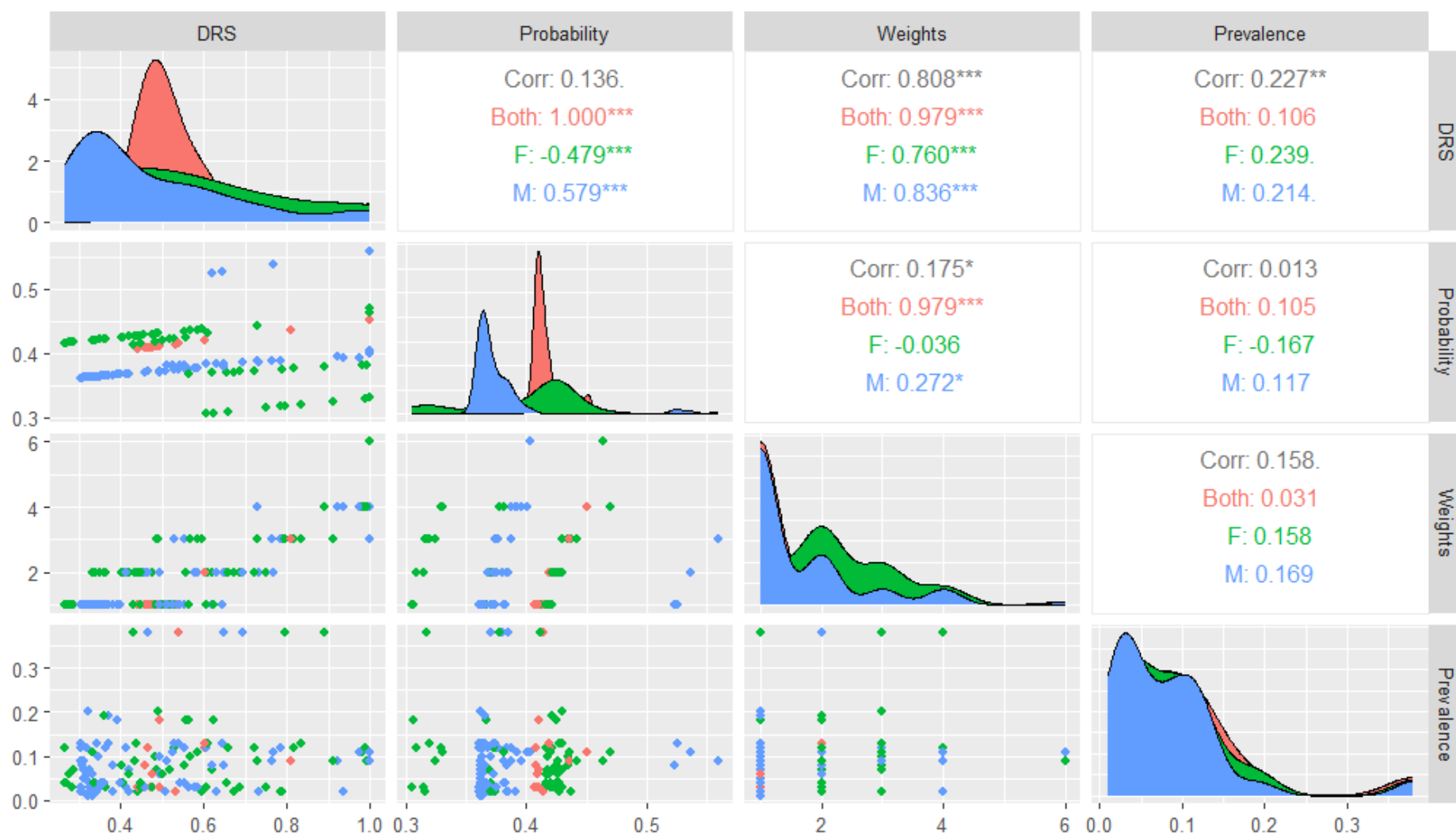

Supplementary figure 18. Pairwise correlation plots by sex – Wave 3, Sweden. *Disease Risk Score = DRS*

Supplement: Supplementary file 1 [file Data_Sheet_1.zip › Image 18.pdf]

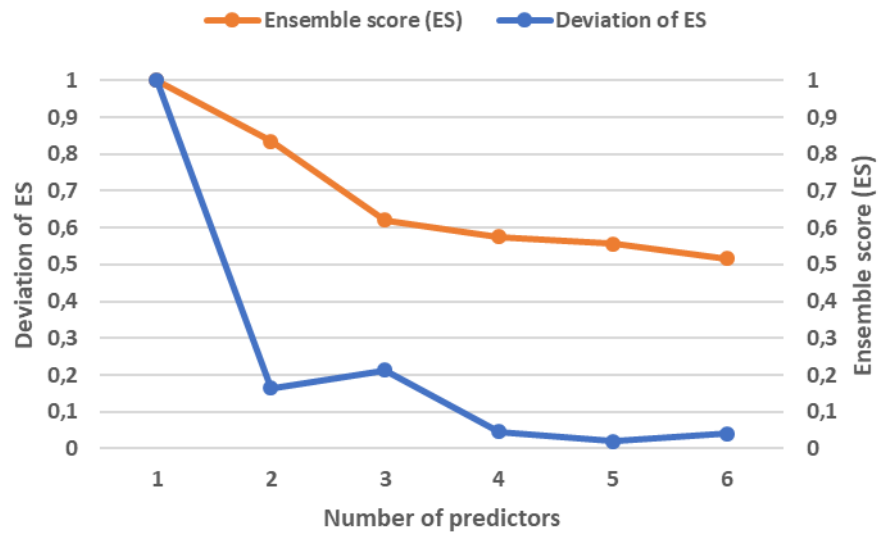

**Supplementary figure 3.** Deviation of the ensemble score – Overall period - Hospitalization.

Supplement: Supplementary file 1 [file Data_Sheet_1.zip › Image 3.pdf]

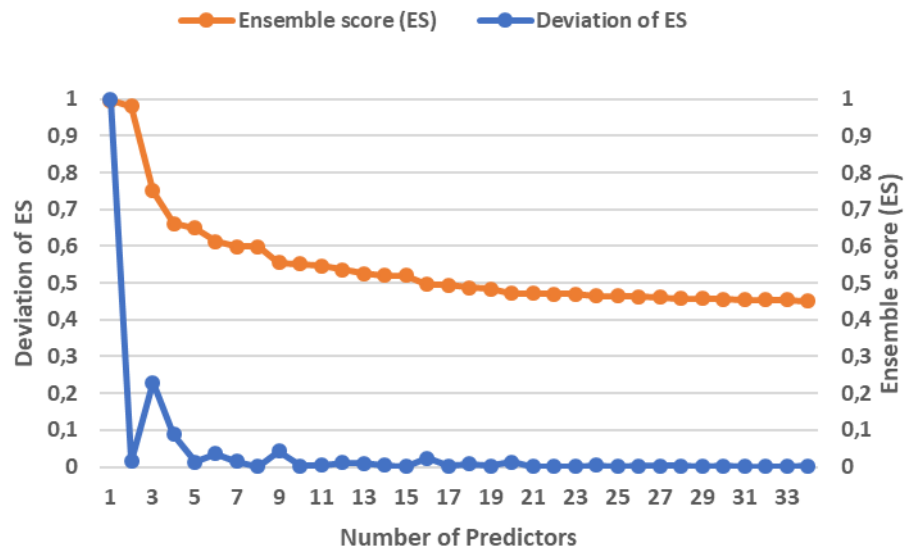

**Supplementary figure 4.** Deviation of the ensemble score – Wave 1 period - Hospitalization.

Supplement: Supplementary file 1 [file Data_Sheet_1.zip › Image 4.pdf]

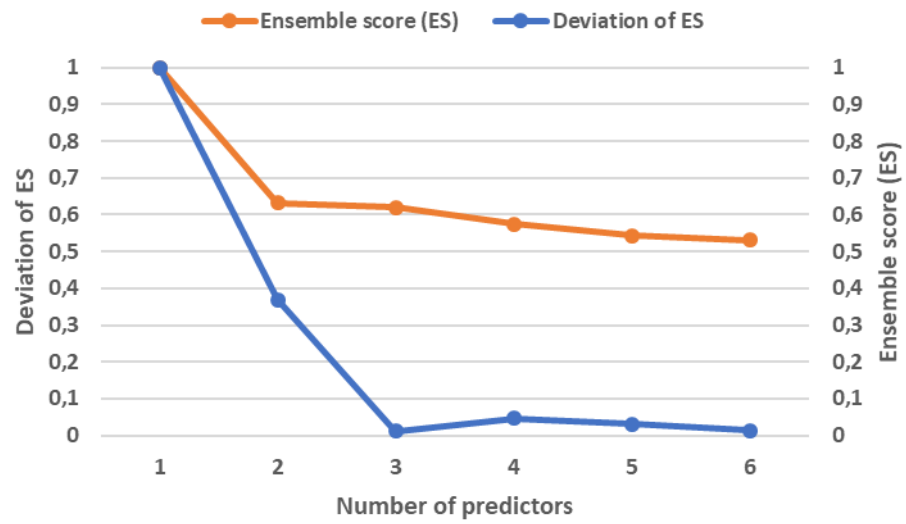

**Supplementary figure 5.** Deviation of the ensemble score – Wave 2 period - Hospitalization.

Supplement: Supplementary file 1 [file Data_Sheet_1.zip › Image 5.pdf]

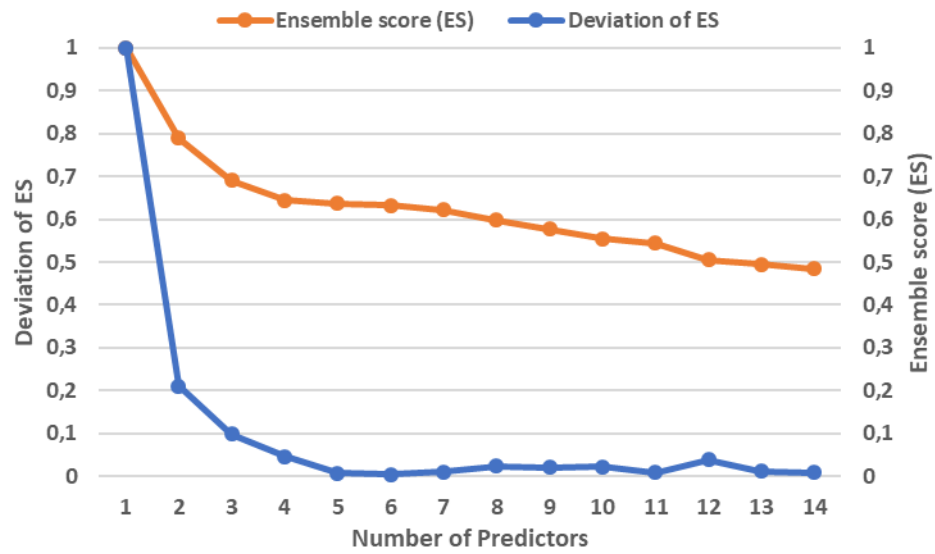

**Supplementary figure 6.** Deviation of the ensemble score – Wave 3 period - Hospitalization.

Supplement: Supplementary file 1 [file Data_Sheet_1.zip › Image 6.pdf]

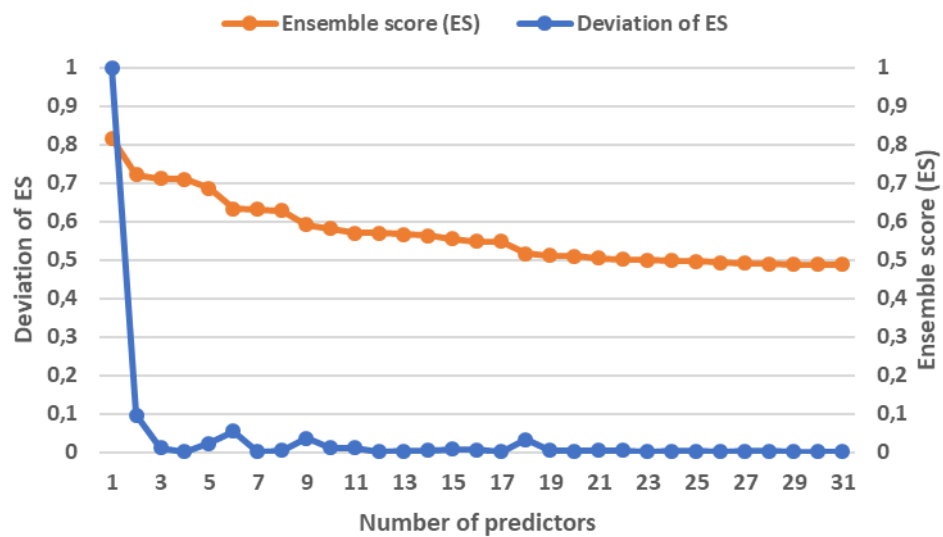

**Supplementary figure 7.** Deviation of the ensemble score – Overall period - Mortality.

Supplement: Supplementary file 1 [file Data_Sheet_1.zip › Image 7.pdf]

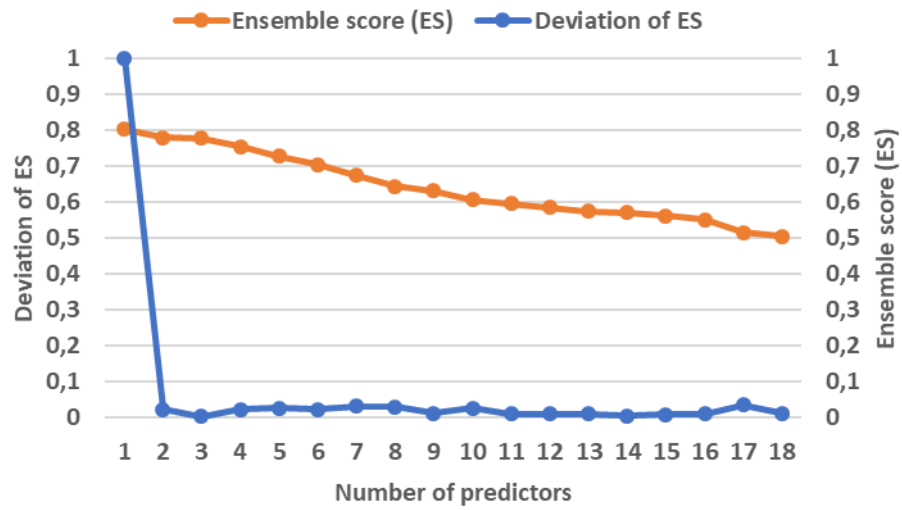

**Supplementary figure 8.** Deviation of the ensemble score – Wave 1 period - Mortality.

Supplement: Supplementary file 1 [file Data_Sheet_1.zip › Image 8.pdf]

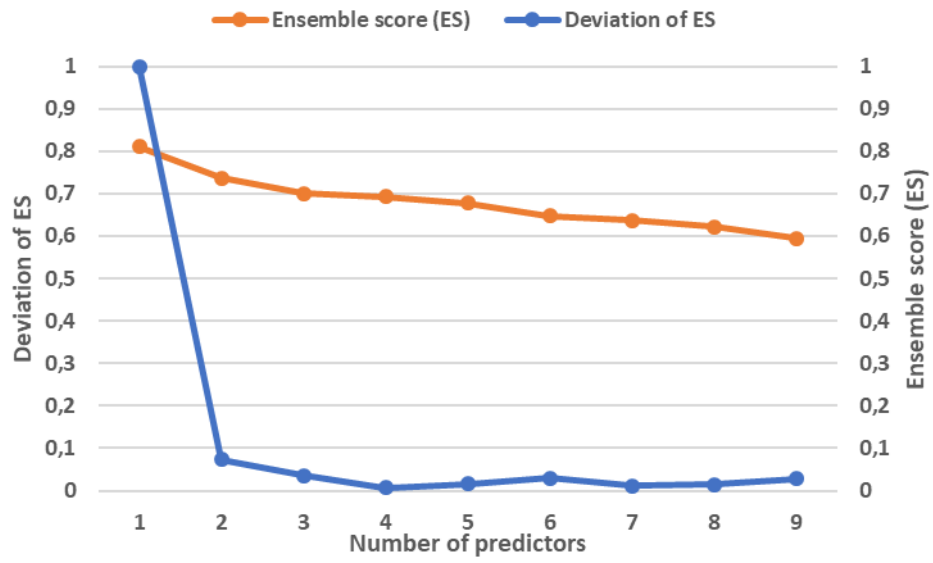

**Supplementary figure 9.** Deviation of the ensemble score – Wave 2 period - Mortality.

Supplement: Supplementary file 1 [file Data_Sheet_1.zip › Image 9.pdf]
